# Symplasmic phloem loading and subcellular transport in storage roots are key factors for carbon allocation in cassava

**DOI:** 10.1093/plphys/kiae298

**Published:** 2024-05-22

**Authors:** David Rüscher, Viktoriya V Vasina, Jan Knoblauch, Leo Bellin, Benjamin Pommerrenig, Saleh Alseekh, Alisdair R Fernie, H Ekkehard Neuhaus, Michael Knoblauch, Uwe Sonnewald, Wolfgang Zierer

**Affiliations:** Division of Biochemistry, Department of Biology, Friedrich-Alexander-University Erlangen-Nuremberg, Staudtstrasse 5, 91058 Erlangen, Germany; School of Biological Sciences, Washington State University, Pullman, WA 99163, USA; School of Biological Sciences, Washington State University, Pullman, WA 99163, USA; Division of Plant Physiology, Department of Biology, University of Kaiserslautern-Landau (RPTU), Erwin-Schrödinger-Str. 22, 67663 Kaiserslautern, Germany; Division of Plant Physiology, Department of Biology, University of Kaiserslautern-Landau (RPTU), Erwin-Schrödinger-Str. 22, 67663 Kaiserslautern, Germany; Division of Central Metabolism, Max-Planck-Institute of Molecular Plant Physiology, Am Mühlenberg 1, 14476 Potsdam, Germany; Division of Central Metabolism, Max-Planck-Institute of Molecular Plant Physiology, Am Mühlenberg 1, 14476 Potsdam, Germany; Division of Plant Physiology, Department of Biology, University of Kaiserslautern-Landau (RPTU), Erwin-Schrödinger-Str. 22, 67663 Kaiserslautern, Germany; School of Biological Sciences, Washington State University, Pullman, WA 99163, USA; Division of Biochemistry, Department of Biology, Friedrich-Alexander-University Erlangen-Nuremberg, Staudtstrasse 5, 91058 Erlangen, Germany; Division of Biochemistry, Department of Biology, Friedrich-Alexander-University Erlangen-Nuremberg, Staudtstrasse 5, 91058 Erlangen, Germany

## Abstract

Cassava (*Manihot esculenta*) is a deciduous woody perennial shrub that stores large amounts of carbon and water in its storage roots. Previous studies have shown that assimilating unloading into storage roots happens symplasmically once secondary anatomy is established. However, mechanisms controlling phloem loading and overall carbon partitioning to different cassava tissues remain unclear. Here, we used a combination of histological, transcriptional, and biochemical analyses on different cassava tissues and at different timepoints to better understand source–sink carbon allocation. We found that cassava likely utilizes a predominantly passive symplasmic phloem loading strategy, indicated by the lack of expression of genes coding for key players of sucrose transport, the existence of branched plasmodesmata in the companion cell/bundle sheath interface of minor leaf veins, and very high leaf sucrose concentrations. Furthermore, we showed that tissue-specific changes in anatomy and non-structural carbohydrate contents are associated with tissue-specific modification in gene expression for sucrose cleavage/synthesis, as well as subcellular compartmentalization of sugars. Overall, our data suggest that carbon allocation during storage root filling is mostly facilitated symplasmically and is likely mostly regulated by local tissue demand and subcellular compartmentalization.

## Teaser

Cassava plants contain a largely passive symplasmic phloem system in which carbon allocation to storage tissues corresponds with tissue-specific changes in sucrose cleavage and sub-cellular compartmentalization.

## Introduction

Cassava (*Manihot esculenta*) is an important crop in Sub-Saharan Africa, South America, and Southeast Asia. Its starchy storage roots are crucial for food security, especially in Sub-Saharan Africa, where over 60% of the global storage root production is realized (FAOSTAT 2022). Despite an overall increase in worldwide cassava production, yields in Sub-Saharan Africa are largely stagnant (FAOSTAT 2022). This, combined with an increasing population and a decrease in the productivity of other crops due to the advancing climate change, makes a sustainable increase in cassava storage root yield paramount for future food security. To this end, advances in cassava breeding and biotechnology are important, which require a better understanding of its physiology.

Cassava is a woody perennial originating from the tropical savanna regions of South America, where it was cultivated over 6,000 yr ago (Olsen and Schaal 1999). Perennial shrubs and trees in these areas follow a deciduous life cycle, losing their leaves during the dry season and regrowing them during the rainy season (Opler et al. 1976). This growth pattern makes effective storage of carbon and water important. Woody plants can store impressive amounts of non-structural carbohydrates (NSC), such as starch, in their trunks and roots (Loescher et al. 1990; Furze et al. 2018). Cassava, in addition to its stem storage tissues, possesses dedicated storage roots that support the accumulation of large amounts of carbon and water. Storage roots initially develop from fibrous roots by initiating secondary growth and subsequently producing large amounts of storage parenchyma cells (Chaweewan and Taylor 2015; Rüscher et al. 2021). This process of lateral size increase and starch filling is referred to as bulking, which is associated with a pronounced accumulation of carbon compounds (Rüscher et al. 2021). Through the activity of a single vascular cambium in the storage root, large amounts of storage parenchyma cells are produced and quickly filled with high levels of starch, in addition to high levels of free amino acids, sucrose, and hexoses (Rüscher et al. 2021). Previous studies have shown that similar to other woody species, phloem unloading in the secondary vasculature of cassava occurs largely symplasmically (Mehdi et al. 2019; Pan et al. 2021). However, it is not clear how assimilates are partitioned between leaves, stems, and storage roots, and how this partitioning is regulated throughout storage root development. However, this process is critical because ineffective carbon flux to storage roots negatively affects yield (Ruiz-Vera et al. 2020; Chiewchankaset et al. 2022). Similarly, the mechanism by which assimilates enter the phloem system in the first place remains unclear to date.

To gain greater insight into the processes regulating carbon allocation and storage in cassava, different cassava tissues and time points were analyzed in terms of their anatomical, transcriptional, and metabolic characteristics. The obtained data indicate that cassava predominantly utilizes a passive symplasmic phloem loading strategy, a conclusion that was further supported using electron microscopy. Furthermore, the importance of local sink strength and intercellular compartmentation of sugars in the storage root is highlighted and supported both by transcriptional analyses and metabolite measurements.

## Results

### Changes in plant anatomy and NSC content during root bulking

**Figure 1.**
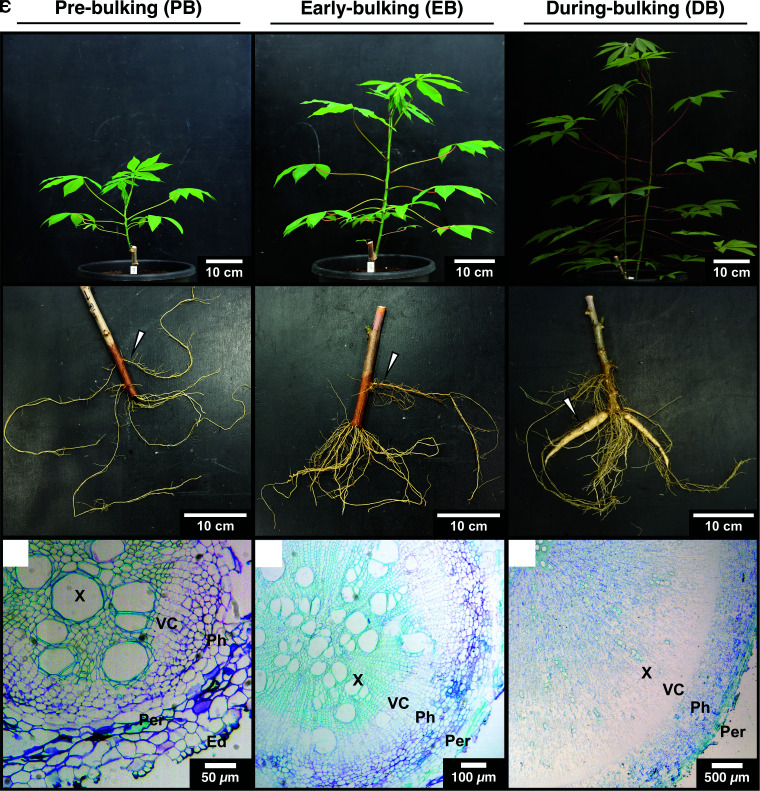
Early development of glasshouse-grown cassava. **A)** to **C)** Pre-bulking ∼30 to 38 dap. **D)** to **F)** Early bulking ∼42 to 51 dap. **G)** to **I)** During bulking ∼60 dap. **A)**, **D)**, and **G)** Shoots. **B)**, **E)**, and **H)** Root stocks. **C)**, **F)**, and **I)** Micrographs of paraffin-embedded storage roots stained with toluidine blue. Arrows in **B)**, **E)**, and **H)** depict the thickest nodal root, which was used for the determination of the developmental stage. Ed, epidermis; Per, periderm; Ph, phloem; Pi, pith; VC, vascular cambium; X, xylem.

Source leaves, stem tissues, fibrous roots, and storage roots at distinct stages of storage root development were analyzed for their anatomy and NSCs. Following earlier studies, we chose the timepoint of initial storage root vascular cambium formation [“pre-bulking,” PB; 30 to 38 days after planting (dap)], the initial expansion of the root xylem via woody tissue deposition (“early-bulking,” EB; 42 to 51 dap), and the stage of storage parenchyma formation (“during bulking,” DB; ∼60 dap) in the storage root (Fig. 1).

While young cassava plants (Fig. 1A) showed no visually distinguishable roots (Fig. 1B) with primary root vascular anatomy (Fig. 1C), slightly older plants (Fig. 1D) already displayed some clearly thickened roots (Fig. 1E), transitioning to secondary root vascular anatomy, indicated by the presence of vascular cambium, yet without storage parenchyma formation (Fig. 1F). At around 60 dap, the plants had grown considerably (Fig. 1G) and had clearly thickening storage roots (Fig. 1H), characterized by a fully annular vascular cambium producing many xylem parenchyma cells (Fig. 1I). Numerous xylem rays (every second to fourth cell layer) were already established during the transition from primary to secondary anatomy at PB (Fig. 1C). During PB and EB roots did produce many xylem fibers and complex secondary tracheary elements (Fig. 1, C and F), while the final storage root did only produce very few such lignified cells and mostly simple tracheary elements (Fig. 1I).

**Figure 2.**
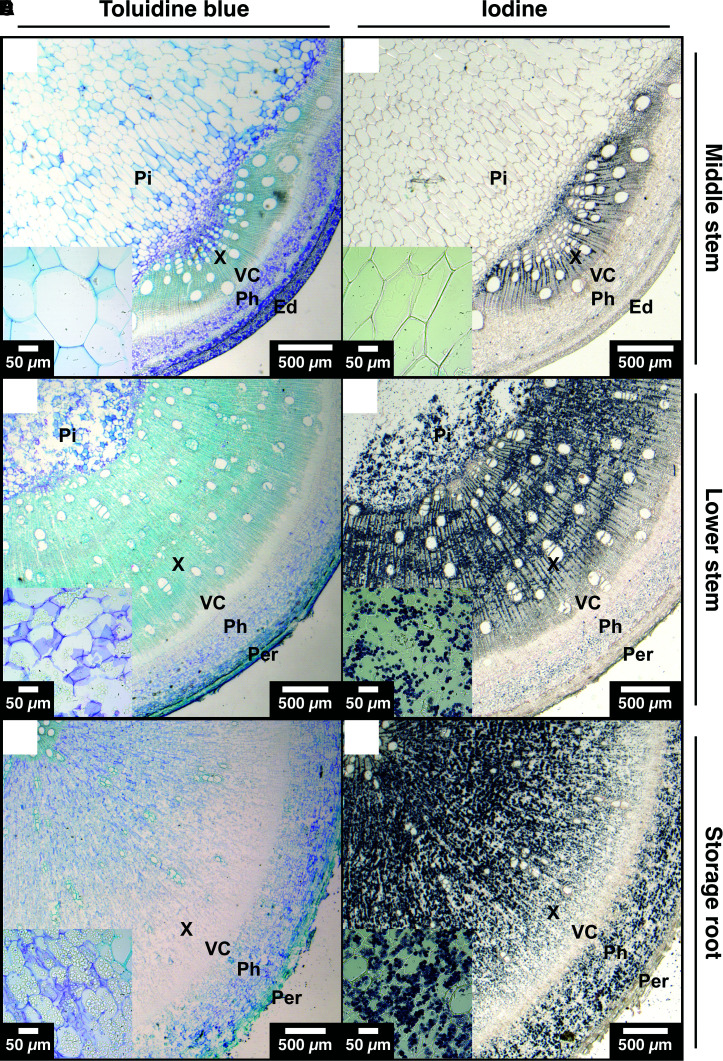
Comparison of cassava stems and storage roots DB. Image pairs of cross-sections stained with either toluidine blue (left column) or iodine (right column) from paraffin-embedded green middle stem **A)** and **D)**, brown lower stem **B)** and **E)**, or storage root **C)** and **F)** samples. Image in **C)** and Fig. 1I are identical. Inlays show a micrograph of pith parenchyma of the stem **A)**, **B)**, **D)**, and **E)**, or xylem storage parenchyma of the storage root **C)** and **F)**. Ed, epidermis; Per, periderm; Ph, phloem; Pi, pith; VC, vascular cambium; X, xylem.

**Figure 3.**
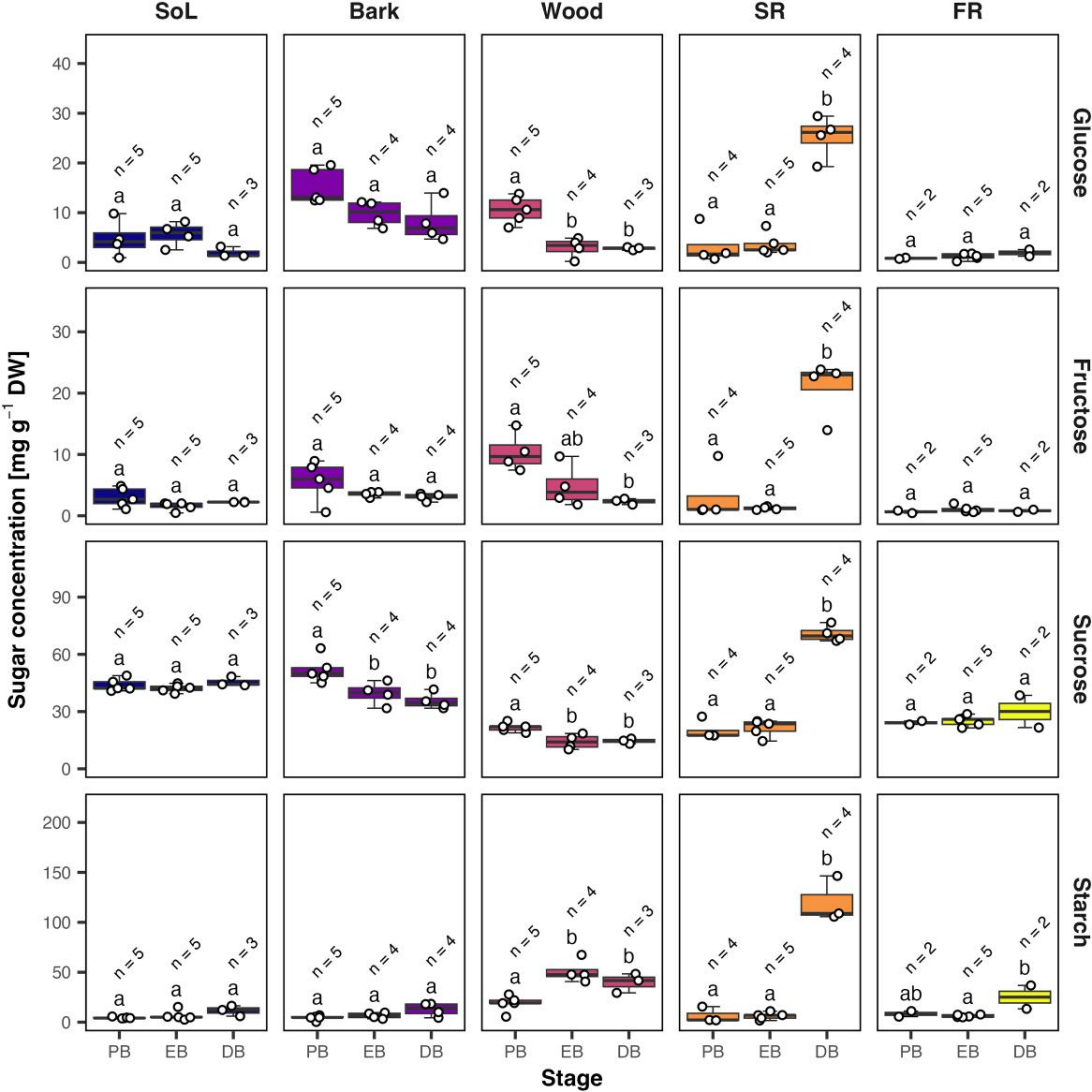
Abundance of major NSC in different casava tissues across developmental stages. Rows from top to bottom show glucose, fructose, sucrose, and starch content in milligrams per gram of dry weight as scatter plots (white dots) and standard boxplots (line: median, box: Quartiles 1 to 3, whiskers: minimum and maximum value excluding outliers). Starch content is shown as the amount of glucose units after amylase/amyloglucosidase digestion. Columns and colors indicate different tissues. A generalized linear model was fitted on the raw values versus the stage for each tissue. Letters show groups of significance within each panel (Tukey HSD *P* ≤ 0.05) of models with an FDR-adjusted *P*-value < 0.05 in an ANOVA. PB, pre-bulking; EB, early-bulking; DB, during-bulking; FR, fibrous root; SoL, source leave; SR, storage root.

Adult stems and storage roots mainly differed in the distribution of woody tissue in the xylem and phloem area. The storage root was dominated by parenchyma cells and did not produce many tracheary elements or fibers (Fig. 2C), while the lower stem displayed the opposite morphology (Fig. 2B). Using iodine staining, starch could be detected in the storage root in almost every cell outside the vascular cambium (Fig. 2F). The stem showed iodine staining both in the xylem rays and the axial parenchyma cells connecting them (Fig. 2, D and E). Interestingly, the pith tissue of the adult stem was also parenchymatic and contained many starch granules (Fig. 2E). This pith storage parenchyma was only found in the oldest stem parts, whereas younger stem regions showed larger cells with small secondary cell walls and no starch granules (Fig. 2, A and D). At PB, most of the lower stem wood was comprised of storage parenchyma cells in the pith tissue, while the ratio of starchy to woody tissue decreased with time (Supplementary Fig. S1). This opposing behavior of storage root and wood was further visible in the abundance of major NSC (Fig. 3 and Supplementary Data Set 1).

Soluble sugars in the storage root accumulated upon root bulking (DB), while they decreased in the stem wood, indicating a change in carbon allocation behavior. Starch levels initially increased in the stem xylem between PB and EB, but strongly increased at DB in storage root. In accordance with an iodine staining (Supplementary Fig. S1), starch content was highest in the wood before root bulking (Fig. 3). In bark tissue, only a slight decrease was measured for the soluble sugars, which was significant only in the case of sucrose (Fig. 3). No changes in the soluble sugar contents of the leaves between the different timepoints were apparent. In both leaf and bark tissues, starch increased slightly at DB, but this was not statistically significant (Fig. 3). NSC levels of the fibrous root were largely invariant, but a small increase in starch content was also observed at DB.

### Generating a source–sink transcriptome and phylogenies of selected gene families

To gain insight into the transcriptional changes occurring in the different cassava tissues over time, 5 cassava plants were harvested at 3 different timepoints and the developmental stage of each harvested plant was confirmed by microscopy of the storage root. Source leaves, the bark and wood of the oldest stem parts, fibrous roots, and storage root were sampled and their respective mRNAs were sequenced.

**Figure 4.**
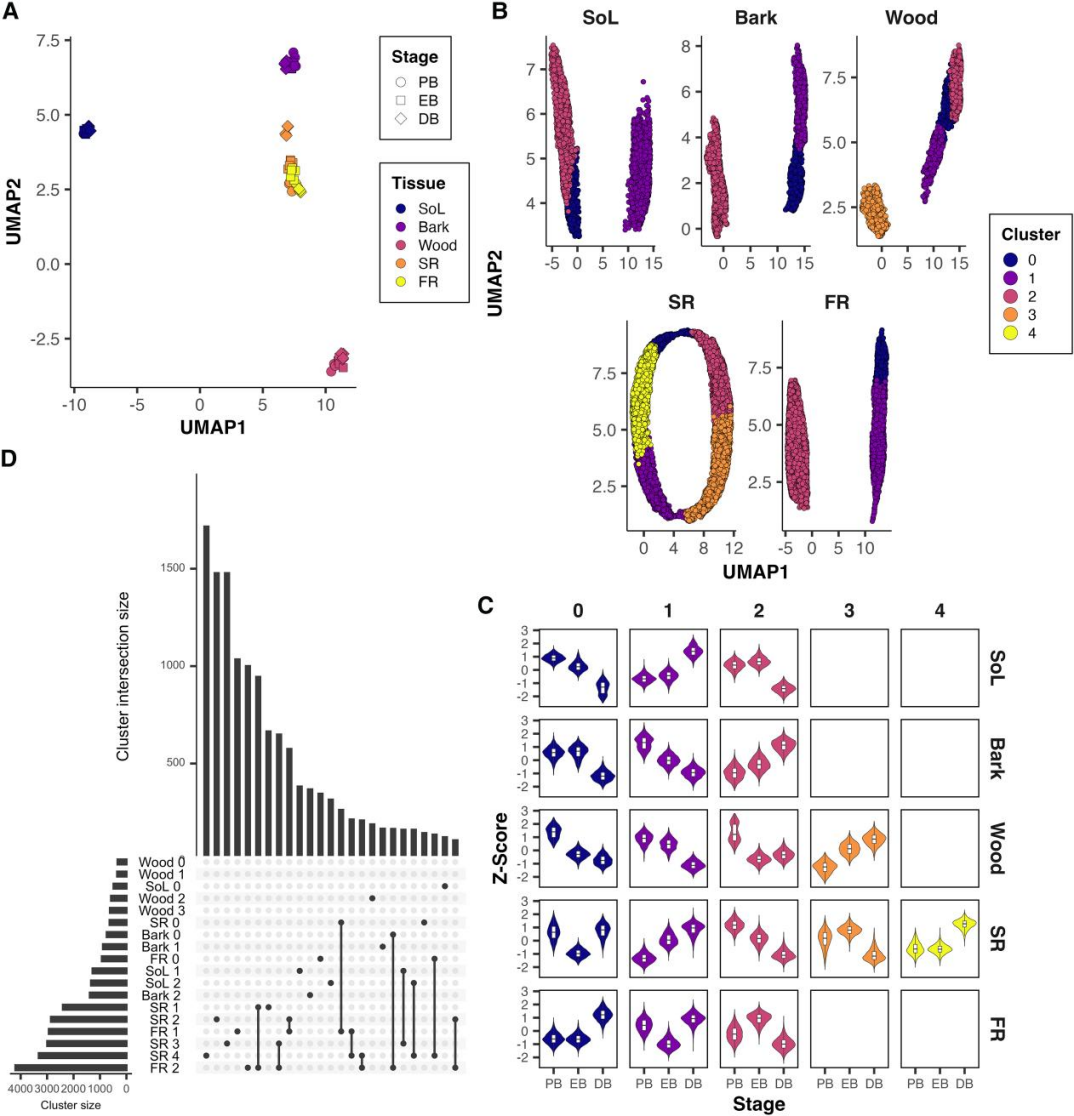
Basic description of the generated cassava transcriptome data. **A)** 2D visualization of samples based on all expressed transcripts (normalized counts >50) using a dimensional reduction projection (UMAP). Counts were normalized using the DESeq2 algorithm. **B)** Visualization of a co-regulation network analysis for DEGs [*P*-adjusted (FDR) < 0.001] within a tissue using a UMAP projection. Each point represents a single gene across all samples. Colors indicate the different clusters obtained through community detection. **C)** Expression profiles of all data points for each gene within a cluster (columns) within each tissue (rows) shown as violin plots. **D)** UpSet plot depicting cluster size (horizontal bars) and size of the 25 largest intersections (vertical bars). The intersection is indicated as connected dots below the vertical bar graph. FR, fibrous root; SoL, source leave; SR, storage root; UMAP, Uniform Manifold Approximation and Projection.

In a dimensional reduction projection of the RNA-Seq data (Uniform Manifold Approximation and Projection (UMAP); Fig. 4A), the samples mainly clustered according to their tissue, but some differences between stages could be observed. In particular, the storage root samples of the DB stage were distant from PB and EB, which highlights the strong changes the root underwent during the bulking process. Differentially expressed genes (DEGs) were clustered according to their expression profiles in each tissue (Fig. 4, B and C and Supplementary Data Set 2). The majority of genes displayed significant expression changes in the storage root (12,188), while comparably few genes were differentially expressed in source leaf (3,098), bark (3,008), or wood (1,882) tissue. In total, 8,051 DEGs were observed in fibrous root samples, but only a few showed higher expression in DB compared to the other stages (cluster FR 0). Nevertheless, some genes did overlap with the corresponding storage root cluster (SR 4). These genes were involved in starch metabolism, which was in line with the slight increase in starch levels also observed in fibrous root at DB (Fig. 3). However, the overlap between storage root and fibrous root genes with the same expression profile was small. A total of 7,126 DEGs of the fibrous root were part of FR 1 or 2, which showed a drop or rise in expression at EB (Fig. 4D), something that was not found in the storage root. Four out of 5 storage root clusters (SR 1 to 4) were roughly the same size with 2,400 to 3,312 DEGs. SR 2 and 3 included genes that were generally lower expressed in later stages, while SR 1 and 4 displayed the opposite behavior.

To specifically study the expression of genes involved in phloem loading, carbon transport, and unloading, as well as subcellular carbon and water transport, maximum likelihood trees of target gene families (Table 1) from thale cress (*Arabidopsis thaliana*), black cottonwood (*Populus trichocarpa*), and *cassava* were produced (Supplementary Files S1 to S11), cassava genes were annotated accordingly (Supplementary Data Set 3), and the tissue-specific expression of these genes was analyzed.

**Table 1.** Annotated gene families

Family	Number of genes		
	Cassava	Black cottonwood	Thale cress
*AATP*	2	2	2
*Acid-INV*	9	8	8
*A/N-INV*	11	12	9
*AQP*	43	55	35
*MST*	64	66	53
*PPase*	7	5	3
*SUS/SPS*	12	14	10
*SUT*	5	5	9
*SWEET*	28	26	17
*TPT*	50	54	50
*V-ATPase*	35	25	28

*AATP*, *ATP:ADP ANTIPORTER*; *Acid-INV*, *ACID INVERTASE*; *A/N-INV*, *ALKALINE/NEUTRAL INVERTASE*; *MST*, *MONOSACCHARIDE TRANSPORTER*; *PPase*, *PYROPHOSPHATASE*; *SUS/SPS*, *SUCROSE SYNTHASE/SUCROSE PHOSPHATE SYNTHASE*; *SUT*, *SUCROSE TRANSPORTER*; *SWEET*, *SUGARS WILL EVENTUALLY BE EXPORTED TRANSPORTER*; *TPT*, *TRIOSE PHOSPHATE TRANSLOCATOR*; *V-ATPase*, *VACUOLAR ATPase*.

### Phloem loading and unloading

**Figure 5.**
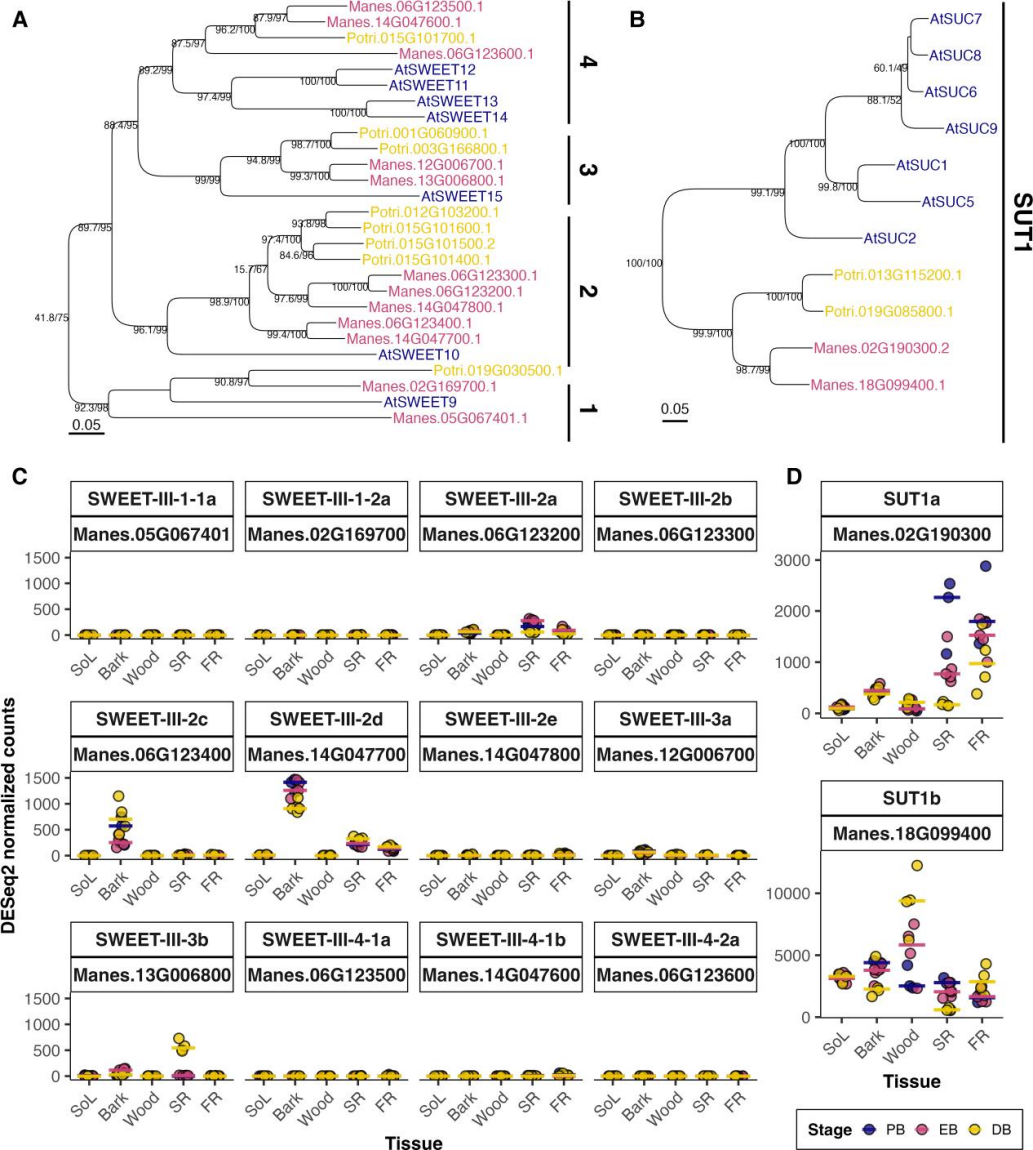
Annotation and expression profiling of cassava *SWEET III* and *SUT1* genes. **A)** and **B)** Subtrees of **A)** sucrose transporting clade III *SWEET* and **B)** *SUT1* genes in *A. thaliana*, *P. trichocarpa*, and *M. esculenta*. Subtrees were taken from midpoint-rooted maximum likelihood trees of full-length CDS sequences (see Supplementary data). Branch labels indicate ultra-fast bootstrap support in percent and shlrt results. Trees were generated using IQTree. Vertical bars and names describe the sub-clades. **C)** and **D)** Scatterplot displaying the expression levels of cassava **C)** *SWEET* clade III and **D)** *SUT1* orthologs. Colors indicate the developmental stage. Horizontal lines show the median per developmental stage. Panel headers describe the here given name and locus identifier, respectively. The bottom right legend is shared between **C)** and **B)**. *n* = 12 or 11 (SoL, Wood). FR, fibrous root; SoL, source leave; SR, storage root.

Phloem loading of sucrose is heterogeneous in nature (Slewinski et al. 2013) but can generally be split into 3 distinct strategies: (1) active apoplasmic, (2) passive symplasmic, and (3) active symplasmic/polymer trapping (Rennie and Turgeon 2009). To this end, phylogenetic trees of *SUGARS WILL EVENTUALLY BE EXPORTED TRANSPORTER* (*SWEET*) (Fig. 5A) and *SUCROSE TRANSPORTER* (*SUC/SUT*) (Fig. 5B) genes were generated and their expression profiled. Active apoplasmic loading requires facilitated export of sucrose from the mesophyll into the apoplast via SWEET11/12 (Chen et al. 2012) and active import via SUT1/SUC2 into the companion cell (CC) (Gottwald et al. 2000). Of the annotated SWEET encoding genes (Fig. 5A), no expression of the *AtSWEET11-14* orthogroup nor any other sucrose transporting clade III *SWEET* was found in the leaves of cassava (Fig. 5C). Furthermore, the expression profile of the *SUT1* orthologs more closely resembled passive symplasmic tree species compared to apoplasmic loaders. For example, in the active apoplasmic loader *A. thaliana*, *AtSUC2/SUT1* is specifically expressed in CC (Truernit and Sauer 1995) and is commonly used to facilitate the CC-specific expression of transgenes, while orthologs in tree species are mainly expressed in the wood in addition to the phloem (Decourteix et al. 2008; Dobbelstein et al. 2019). Expression of both *MeSUT1* orthologs decreased over time in the storage root and bark but increased in the wood (Fig. 5D and Supplementary Data Set 3). *MeSUT1b* had much higher expression than *MeSUT1a* and was most abundant in woody tissues. *MeSUT1a* had its highest expression in fibrous root and young storage root. However, *MeSUT1b* expression was still present in the source leaf.

**Figure 6.**
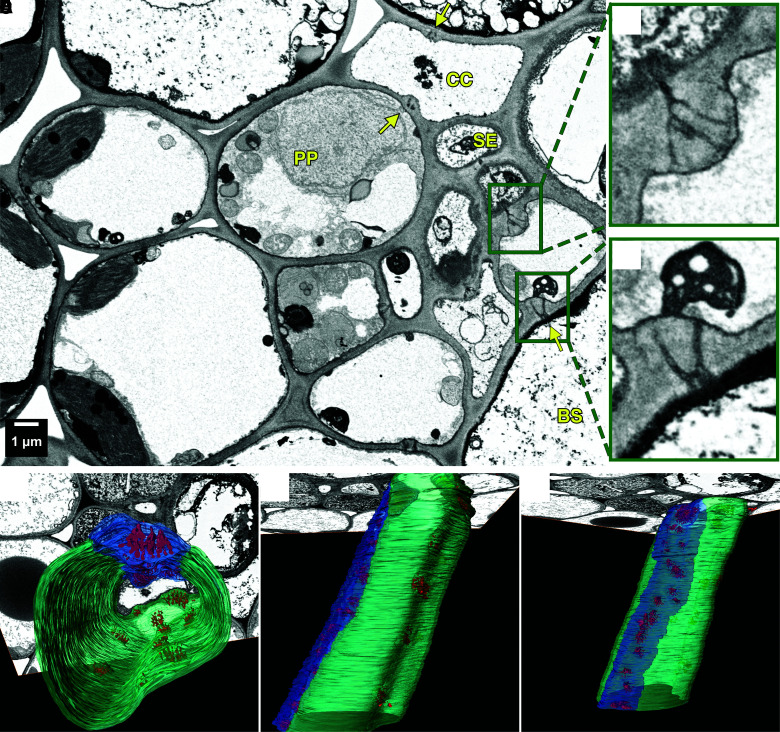
SBFSEM of a cassava minor vein. **A)** A single 2D image of a 3D stack of a cassava minor vein taken by SBFSEM. Arrows indicate branched plasmodesmata between BS/phloem parenchyma and CCs. Green zoom-out boxes show **B)** a pore plasmodesma between an SE and a CC and **C)** a branched plasmodesma between a BS and a CC. **D)** to **F)** 3D reconstruction of a minor vein CC from the same stack as shown in 2D in **A)**. Blue shows the interface of CC and SE containing pore plasmodesmata (red). Cyan highlights the cell wall interface between the CC and BS containing branched plasmodesmata (red). The differences in plasmodesma density between the individual interfaces is apparent. BS, bundle sheath; CC, companion cell; PP, phloem parenchyma; SE, sieve element.

To gather more information about the loading type of cassava, serial block face scanning electron microscopy (SBFSEM) of cassava minor veins was performed to analyze the presence and anatomy of plasmodesmata in the CC/bundle sheath (BS) interface (Fig. 6, A to C). Several branched plasmodesmata were present at the CC/BS interface (Fig. 6, A and C), characteristic for symplasmic, but not apoplasmic loaders (Haritatos et al. 2000; Rennie and Turgeon 2009), while the CC/sieve element (SE) interface showed the generally typical pore plasmodesmata (Fig. 6B). However, plasmodesmata in cassava minor veins were not found throughout the whole CC/BS interface but were arranged in pith fields made visible through SBFSEM (Fig. 6, D and F and Video 1).

In addition, the sucrose concentration in cassava source leaves is very high with more than 200 mmol l^−1^ at the DB stage (Supplementary Fig. S2 and Supplementary Data Set 4), which is another characteristic of passive symplasmic loaders (Rennie and Turgeon 2009). The highest sucrose concentration reported there was 150 mmol l^−1^ for a passive symplasmic loader. An active polymer trapping mechanism is unlikely, due to the absence of specialized intermediary cells, as well as the lack of raffinose in leaf phloem exudates (Supplementary Fig. S3 and Supplementary Data Set 5) (McCaskill and Turgeon 2007; Rennie and Turgeon 2009).

**Figure 7.**
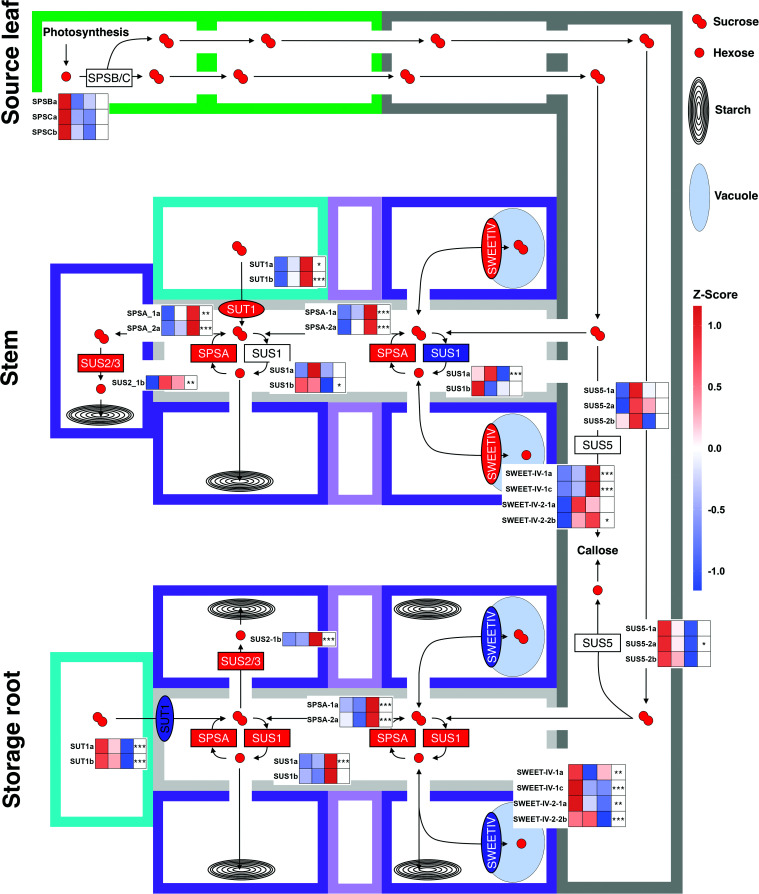
Changes in tissue-specific gene expression for annotated genes in leaf, stem, and storage root. The top, middle, and bottom rows depict the source leave, stem, and storage root of the cassava plant. Colored rectangles display idealized cells: green, leaf mesophyll/BS; dark-gray, CC/SE complex; light-gray, xylem ray; dark purple, parenchyma cell; light purple, vascular cambium; turquoise, tracheary element. Light blue ellipses are vacuoles, concentric ellipses amyloplasts. Smaller rectangles and ellipses indicate enzymes and transport proteins. Proteins which genes show a general upward or downward trend overtime are colored red or blue, respectively. The heatmaps within the cells display the *z*-score of the average log-transformed values per stage within the given tissue. Stem bark and wood is separated by the vascular cambium. Expression in the storage root is for both xylem and phloem tissue. A likelihood-ratio test was performed with the R package DESeq2 using the intercept only as a reduced model. Asterisks indicate significant changes (FDR-adjusted *P*-values; * < 0.05; ** < 0.01; *** < 0.001). Only genes with an average of 50 or higher DESeq2 normalized counts per condition were plotted. SoL, source leave; SR, storage root.

In addition to the expression changes of *SWEETs* and *SUT1* genes (Fig. 5), *SUCROSE-PHOSPHATE SYNTHASE (SPS)* and *SUCROSE SYNTHASE* (*SUS*) genes (Fig. 7) also showed clear tissue-specific expression changes among the selected and annotated genes (Table 1). SUS1/4 proteins have previously been shown to be associated with symplasmic unloading (Zrenner et al. 1995; Bieniawska et al. 2007; Gerber et al. 2014; Mehdi et al. 2019; Yao et al. 2019). Of the *MeSUS1* genes that could be annotated, *MeSUS1a* had the highest transcript levels and was differentially expressed in bark and storage roots. It was grouped into clusters Bark 0 and SR 4 (Fig. 4 and Supplementary Data Set 2), meaning transcript abundance decreased at DB in bark, but increased in storage root (Fig. 7). Overall, *MeSUS1a* was most highly expressed in the wood and adult storage root. *MeSUS1b* showed the same tendencies but did not reach statistical significance, except in source leaf samples, where it decreased at DB (cluster SoL 0). The temporal expression profile of the *MeSUS1* orthologs did not match starch levels, implying a more general role of this isozyme in ensuring carbon availability for the sink tissues as described in poplar (Coleman et al. 2009; Gerber et al. 2014). However, *MeSUS3* did follow starch content more closely within each tissue. In *A. thaliana*, SUS3 is associated with seed endosperm tissue, which could imply a role in storage (Yao et al. 2019). In addition to *MeSUS1*, *MeSPSA* genes increased in transcript abundance not only in bark and storage root (Fig. 7) but also in the other tissues (Supplementary Data Set 3). Sucrose cycling in sink organs is a commonly observed phenomenon, potentially explaining the changes in SPSA expression (Geigenberger and Stitt 1993; Nguyen-Quoc and Foyer 2001). The other *MeSPSA* gene was similarly expressed across tissues, while transcripts for genes encoding for *MeSPSB/C* proteins were much more abundant in the source leaf, suggesting their involvement in photosynthetic sucrose production (Fig. 7). Another interesting aspect is the tissue-specific regulation of genes encoding for tonoplast localized clade IV SWEET proteins (Klemens et al. 2013; Guo et al. 2014). SWEETIV proteins are involved in wood formation in *A. thaliana* (Aubry et al. 2022). Indeed, increased expression was observed in cassava bark tissue upon increased wood formation at DB and a decrease in *MeSWEETIV* expression was observed in storage root when barely any wood is formed. Generally, changes in the expression of tonoplast sugar transporters were among the more obvious changes in the sugar-related transcripts of the DEGs found in the storage root.

### Compartmentation of metabolites in the bulking storage root

Most known genes involved in vacuolar sugar import were more highly expressed in later stages or at DB. These include all 4 *TONOPLAST SUGAR TRANSPORTER* (*MeTST*)*1/2* that were all part of cluster SR 4. The only expressed *TST3* orthologue (*MeTST3b*) increased throughout bulking (cluster SR 1). TSTs are H^+^/hexose antiporters (Wormit et al. 2006; Wingenter et al. 2010), but TST1 and TST2 can also facilitate the transport of sucrose into the vacuole (Schulz et al. 2011; Jung et al. 2015).

Other genes grouped in clusters SR 1 and 4 include 4 of 5 *VACUOLAR PYROPHOSPHATASE* (*MeAVP*)*1*, as well as 26 of 35 genes encoding for MeV-ATPase subunits. Both of these proteins allow for energization of the vacuole (Dietz et al. 2001; Gaxiola et al. 2001). The remaining V-ATPase orthologs were either not clustered or not expressed in the storage root.

Conversely, transcripts of proteins involved in vacuolar export such as the H^+^/sucrose symporter MeSUT4b (Schneider et al. 2012), as well as all 3 EARLY RESPONSE TO DEHYDRATION 6 (MeERD6) proteins (Kiyosue et al. 1998), and 2 of 3 MeEDL4/ERDL6 orthologs (Poschet et al. 2011) decreased throughout bulking (cluster SR 2 or 3). ERD6- and ERD6-like proteins are part of the MST family and facilitate H^+^/hexose symport across the tonoplast (Pommerrenig et al. 2018; Khan et al. 2023).

In addition to NSC content (Fig. 3), metabolite profiles of EB and DB samples were measured. These showed an increase in *myo*-inositol and other putative vacuolar metabolites (Supplementary Data Set 6). INT1 is the only known protein to be able to export *myo*-inositol from the vacuole (Schneider et al. 2008). The sugar alcohol *myo*-inositol and, indeed, all soluble NSCs are used by plants to increase the osmotic potential of the vacuole. Accordingly, changes in the expression of genes relevant to water transport were also observed. Passive transport of water molecules across membranes is enabled by AQUAPORIN (AQP) family proteins. Plasma membrane and tonoplast localized AQPs are called PLASMA MEMBRANE INTRINSIC PROTEINS (PIPs) and TONOPLAST INTRINSIC PROTEINS (TIPs), respectively. In poplar another plasma membrane-localized group of AQPs was identified (uncategorized intrinsic proteins (XIPs); Lopez et al. 2012; Maurel et al. 2015), but, while present, no members of this subfamily were expressed in cassava. However, 9 of 13 expressed PIPs and 5 of 9 expressed TIPs were significantly increased in expression throughout root bulking (cluster SR 1 or 4).

**Figure 8.**
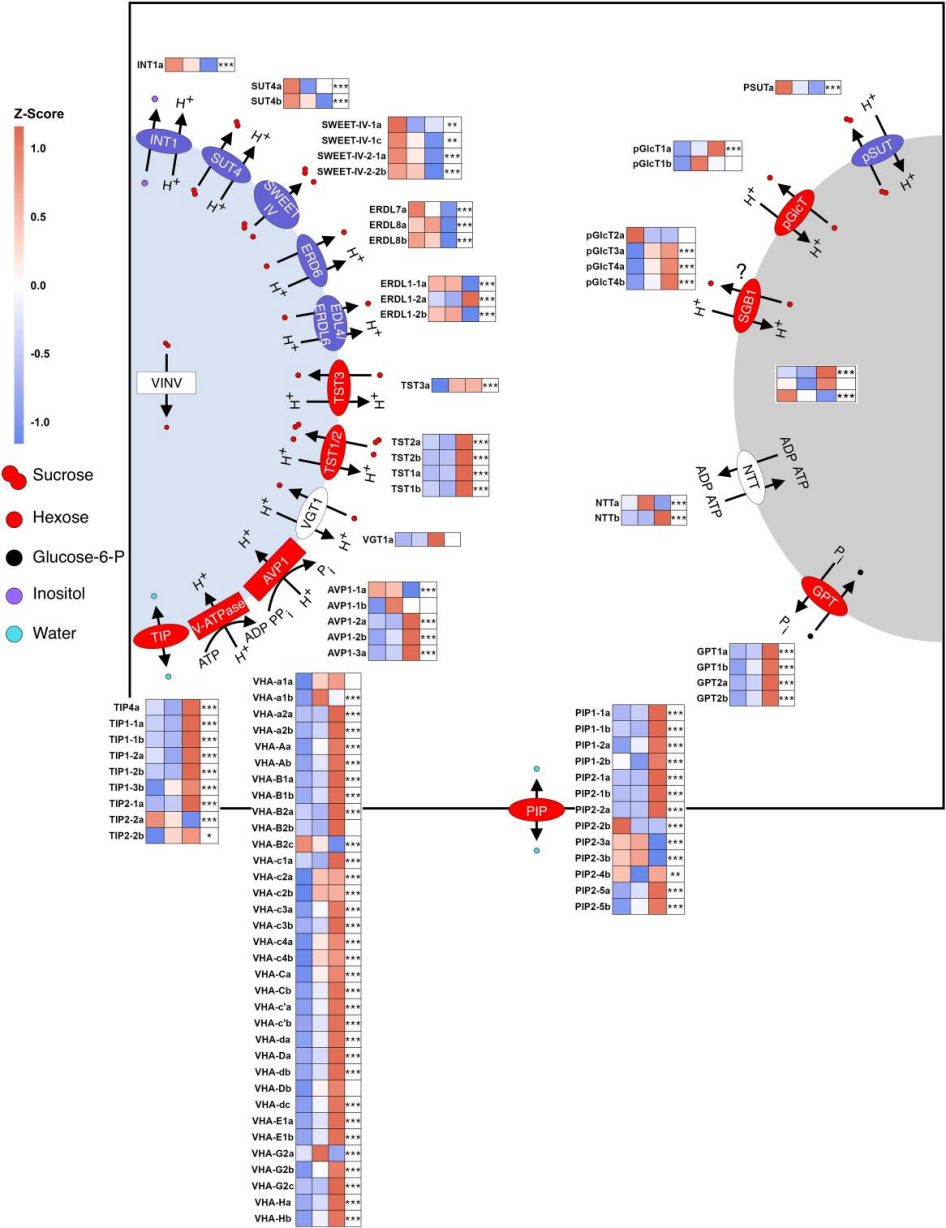
Vacuolar and amyloplast transporters in cassava storage roots throughout bulking. The blue and gray half-circle represent the vacuole and amyloplast, respectively. The black border shows the plasma membrane of a parenchyma cell. Rectangles and ellipses indicate enzymes and transport proteins, respectively. Proteins which genes show a general upward or downward trend overtime are colored red or blue, respectively. Heatmaps show the *z*-score of the average VST value per stage in the storage root of 2 independent experiments. Only genes with >50 normalized counts on average are shown. From left to right: Pre-bulking, ∼30 to 38 dap; Early bulking, ∼42 to 51 dap; and During bulking, ∼60+ dap. A likelihood-ratio test was performed with the R package DESeq2 using the intercept only as a reduced model. Asterisks indicate significant changes (FDR-adjusted *P*-values; * < 0.05; ** < 0.01; *** < 0.001).

Due to the high starch content of storage parenchyma cells, the expression of genes involved in carbon allocation into amyloplasts was also analyzed. Interestingly, all 4 *GLUCOSE-6-PHOSPHATE/PHOSPHATE TRANSLOCATOR* (*GPT*) genes sharply increased in their abundance at DB in storage root (cluster SR 4, Fig. 8). GPT supplies the amyloplast with glucose-6-phosphate (G6P) in exchange for phosphate (Kammerer et al. 1998). The G6P isomer G1P is a precursor to ADP-glucose which is the substrate for starch biosynthesis. During the conversion of ADP-glucose to starch ADP is produced, which is exchanged with ATP by a NUCLEOTIDE TRANSPORTER (NTT) (Reiser et al. 2004). Two orthologs of *NTT* could be annotated in cassava, one was co-expressed with the *GPT* orthologs (Fig. 8). The expression of the only annotated plastid sucrose transporter (*PLASTID SUGAR TRANSPORTER, pSUT*) was decreased upon root bulking, while 1 of 2 *PLASTID GLUCOSE TRANSPORTERS* (*pGlcT*) showed the opposite behavior. Both of these transporter types export their respective substrates from the plastid to the cytosol (Cho et al. 2011; Patzke et al. 2018). While it is not fully clear, where the H^+^/glucose antiporters *SUPPRESSOR OF G PROTEIN BETA1* (*SGB1*) are localized within the cell, they share a phylogenetic group with *pGlcT*. Three of 4 *MeSGB1* orthologs were found in cluster SR 1, as they were higher expressed throughout root bulking.

**Figure 9.**
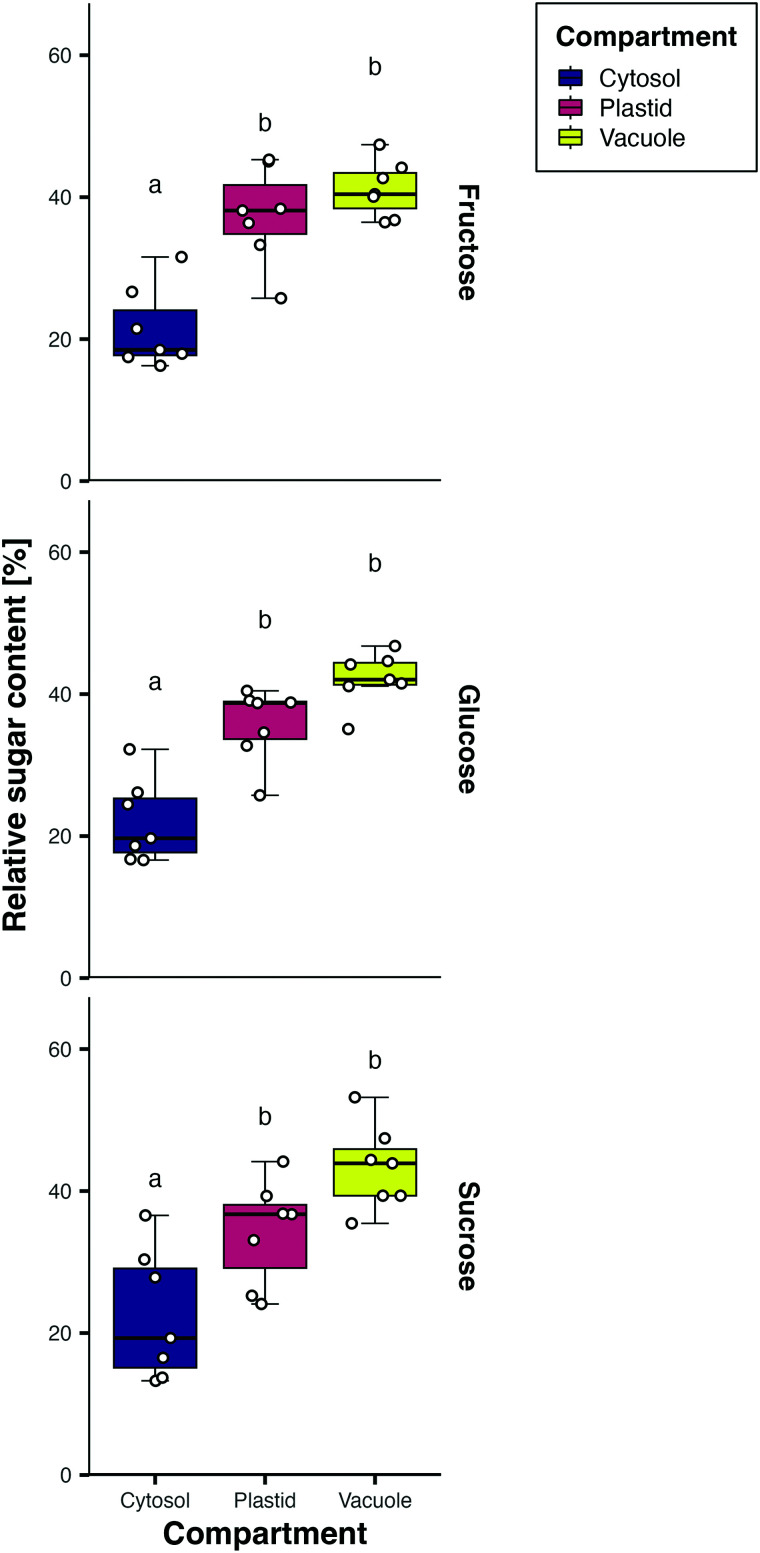
NAF and sugar measurement of adult cassava storage roots. Data are displayed as percent of total sugar content. Samples are independent from the other experiments. Rows from top to bottom show fructose, glucose, and sucrose content. A linear model was fitted using predicting the relative sugar quantities per compartment across 7 replicates. Letters show groups of significance within each facet (Tukey HSD *P* ≤ 0.05) of models with an FDR-adjusted *P*-value < 0.05 in an ANOVA.

To directly measure metabolite compartmentation within the storage root, non-aqueous fractionation (NAF) was performed to compare the NSC content of the cytosol, vacuoles, and plastids (Fig. 9). Unfortunately, only adult storage roots could be measured successfully. In these, glucose, fructose, and sucrose showed the same relative distributions across the different compartments (Fig. 9), but sucrose was much more abundant (Supplementary Data Set 7). As suggested by the changes in gene expression of vacuolar transporters (Fig. 8), NSC abundance was highest in the vacuole with ∼40% of the total content, however, plastids were a close second with around 35%. As expected, most of the sugar accumulating in the storage root was not present in the cytosol but was compartmentalized into other subcellular compartments. The presence of sucrose in amyloplasts was additionally described for potato tubers, however, while a chloroplast transporter capable of transporting sucrose has been identified (Patzke et al. 2018), it is as yet unclear how sucrose enters amyloplasts (Hampp and Schmidt 1976; Gerrits et al. 2001). The presence of hexoses in the plastid can be explained through plastid A/N-INV proteins (Gerrits et al. 2001). These genes were all expressed, but no DEGs were found between the different tissues and timepoints, respectively (Fig. 8). Overall, the data indicate pronounced regulation of subcellular transport during storage root bulking.

## Discussion

### Cassava stems are large carbon sinks with complex development

Stems of any type of plant are no mere highways for metabolite and nutrient transfer but are rather important and complex carbon sinks, especially in woody perennials such as cassava (Furze et al. 2018). Even in the presented short-time glasshouse study, the plant stems contained more than 5% of their total DM as starch, while also producing the necessary secondary cell walls (Fig. 3). The tissue composition of cassava stems also appeared to be connected to the initiation of the storage root (Fig. 2 and Supplementary Fig. S2). The first newly formed stems were largely made up of large pith parenchyma cells, which were filled with starch. These cells apparently reach their full capacity already during the EB stage. Coincidentally, this was around the same time the first starch granules could be found in xylem rays and cortex parenchyma of developing storage roots (Supplementary Fig. S1). Hence, induction of the storage root could function as an “overflow basin” for unmetabolized sugar of the stem, which would explain why root bulking initially occurs proximally to the shoot. It is likely that sugar signaling plays an important role in storage root initiation as starch metabolism in the root occurs earlier than storage parenchyma formation. Stem starch metabolism, however, is not limited to the pith parenchyma but can be observed in xylem rays as well as axial parenchyma, which is the usual xylem parenchyma pattern in different woody species (Sokołowska and Zagórska-Marek 2012; Furze et al. 2018).

### Parenchymatic cells of source and sink tissues are symplasmically connected

Phloem loading can be grouped into 3 categories. Apoplasmic phloem loading is commonly known from herbaceous species such as *A. thaliana*, but also root and tuber crops such as potato, where sucrose is transported from the phloem parenchyma/transfer cells into the surrounding cell wall space by clade III SWEETs (Chen et al. 2010, 2012) and subsequently taken up into phloem CCs by SUT1 proteins. However, most woody plants primarily use a passive symplasmic mode of phloem loading (Rennie and Turgeon 2009; Slewinski et al. 2013; Zhang et al. 2014). Expression of phloem loading *SWEET11/12* can typically only be measured in the leaves and the phloem of apoplasmic loaders (Chen et al. 2012; Abelenda et al. 2019; Doidy et al. 2019) but not in passive loaders (Zhang et al. 2020). Three *SWEET* genes were found in the same orthogroup (*SWEET-III-4*) as *AtSWEET11/12* (Fig. 5A, Supplementary Data Set 3), but none showed expression in any tissue including the source leaf, nor was any other putative sucrose transporting clade III *SWEET* expressed in the source leaf (Fig. 5C). These findings suggest a predominantly symplasmic loading strategy. Additionally, *MeSUT1* expression profiles more closely resembled the pattern of passive symplasmic loading woody perennials, which utilize SUT1 proteins for sucrose uptake from xylem vessels during remobilization, rather than for phloem loading (Loescher et al. 1990; Decourteix et al. 2006). These genes are most highly expressed in bark and wood tissue (Decourteix et al. 2006, 2008; Dobbelstein et al. 2019) and their mRNAs are detectable in xylem parenchyma (Decourteix et al. 2008), but expression is still high in phloem tissue (Schrader et al. 2004). *MeSUT1b*, specifically, was more highly expressed in bark and wood tissue, as well as in the source leaf. However, it showed an opposing expression pattern in wood and storage root coinciding with the ratio of woody xylem to storage parenchyma (Fig. 7), implying an expression in vessel-associated or regular xylem parenchyma cells like in other species and not in the storage parenchyma (Decourteix et al. 2008).

Symplasmic and apoplasmic loaders differ in the number and structure of their plasmodesmata of the CC/BS or CC/PP interface (Turgeon and Medville 2004). Many branched plasmodesmata could be observed in the CC/BS interface of cassava minor veins in SBFSEM images, which is a characteristic of symplasmic loaders, whereas apoplasmic loaders have no or few branched plasmodesmata. Cassava, however, showed an apparently lower frequency than many tree species, as plasmodesmata were only abundant in very few 2D SEM images (Fig. 6, A to C). SBFSEM that allows for continuous imaging of sequential sections through an embedded specimen to generate 3D volumes, however, revealed the presence of highly branched plasmodesmata at this interface (Fig. 6, D to F and Video 1). This highlights the importance of 3D reconstructions when evaluating loading types by anatomical features. Transfer cells, a specialized PP cell type with wall ingrowths toward the CC (Haritatos et al. 2000) that can occur in apoplasmic loaders, were also not found in cassava minor veins, neither were intermediary cells that exist in active symplasmic loaders. Furthermore, sucrose was far more abundant in phloem exudates compared to raffinose (Supplementary Fig. S3). Notably, measured source leaf sucrose concentrations exceed 200 mmol l^−1^ (Supplementary Fig. S2), which is comparable to other known passive loaders (Rennie and Turgeon 2009; Slewinski et al. 2013). In conclusion, the minor vein anatomy, the presence of branched plasmodesmata in the CC–BS interface, high leaf sucrose concentration in the source leaves, as well as the lack of expression of the necessary transporters, makes it likely that cassava predominately utilizes a passive symplasmic phloem loading strategy when transferring carbon from leaf mesophyll cells to sink organs.

However, active sucrose uptake into CCs is still plausible due to the expression of *MeSUT1* orthologs also in phloem-containing tissues in cassava. Yet, without the expression of sucrose-transporting SWEET proteins, it is unclear how much contribution this active mechanism can have to the overall source–sink carbon allocation. It is, however, still possible that active phloem loading serves an important purpose for apoplastic sugar retrieval under specific conditions that are not apparent under our glasshouse study conditions.

Symplasmic unloading strategies are more widespread among different types of plants, varying even between and within a tissue depending on its development (Viola et al. 2001). Arabidopsis roots, potato tubers, as well as the secondary vasculature of woody perennials, including cassava stems and storage roots unload symplasmically (Sokołowska and Zagórska-Marek 2012; Ross-Elliott et al. 2017; Mehdi et al. 2019). Several studies have shown the involvement of SUS proteins in symplasmic unloading processes. SUS proteins can be grouped into 3 phylogenetic clades. The SUS1 clade contains the highly expressed *AtSUS1* and *AtSUS4* genes, which encoded proteins that localize to the CCs (Bieniawska et al. 2007; Yao et al. 2019). However, neither knockout of *AtSUS1* (Barratt et al. 2009), nor knockdown or overexpression of aspen *SUS1* genes alters the macroscopic growth phenotype in glasshouse studies (Coleman et al. 2009; Gerber et al. 2014), likely because symplasmic unloading does not involve CCs at least in *A. thaliana* roots (Ross-Elliott et al. 2017). Beyond Arabidopsis, however, increased SUS activity did lead to increased cellulose content and thickened secondary cell walls in aspen (Coleman et al. 2009), while a decrease reduced the overall carbon availability (Gerber et al. 2014). Furthermore, SUS activity is important for starch production in potato tubers (Geigenberger and Stitt 1993; Zrenner et al. 1995). Both the role of SUS in secondary cell wall formation and starch biosynthesis make *MeSUS1* genes interesting research targets. Two *MeSUS1* genes could be annotated (Supplementary Data Set 3) that were highly expressed with counts in the high ten-thousands. Their expression was high in secondary vasculature, but strongest in wood and bulking storage root. Previous *MeSUS1a* promoter studies also showed the strongest activity in stems and storage roots with notable staining also in transport-associated xylem rays present in both tissues (Zierer et al. 2022). The 2 *MeSUS1* genes showed an increase in transcript abundance DB in storage root but much stronger for *MeSUS1a*, which also decreased at DB in bark, implying a change in sink strength towards the bulking storage root (Fig. 7). In addition to *MeSUS1*, *MeSUS3a* expression was high in the storage root and bark, but also in the wood. Within the wood and the storage root, transcript levels of *MeSUS3a* tightly followed the relative starch content (Figs. 3 and 7). This was not true for *MeSUS1a*, implying a role for *MeSUS3a* in starch biosynthesis. *MeSUS5/6* orthologs were only expressed in transport phloem-containing tissues (bark, storage root, and fibrous root), which fits the observation in *A. thaliana*, where SUS5/6 localize to the SEs where they are involved in callose biosynthesis (Barratt et al. 2009; Yao et al. 2019).

In summary, cassava parenchyma cells in the source leaf, stem, and secondary root tissue seem to form one large symplasmically connected system (Fig. 7). As such, carbon allocation is controlled by the phloem Münch flow and local sink strength might mainly be controlled through growth and carbon storage processes, as well as subcellular carbon compartmentalization.

### Subcellular compartmentation of sugars in the storage root is an important aspect of root bulking

Once the bulking process is initiated, cassava storage roots rapidly increase in diameter. Between the EB and DB stages multiple nodal roots per plant grew from a few millimeters to more than a centimeter in thickness (Fig. 1), while producing over 10% of their total dry biomass as starch (Fig. 3). These changes were accompanied by strong changes in the transcriptome of the storage organ (Fig. 4A), including lowered expression of secondary cell wall producing enzymes, as well as an increase in transcript abundance of starch biosynthesis genes as described previously (Rüscher et al. 2021), but also many proteins involved in compartmentation of sugars and other metabolites (Fig. 8).

The MST gene family includes a plethora of genes encoding for proteins involved in tonoplast or plastid transport (Pommerrenig et al. 2018). While transcripts of proteins involved in vacuolar/plastidic import generally increased when cytosolic sugar levels are high, those involved in vacuolar/plastidic export generally decreased in expression under latter conditions (Wormit et al. 2006; Klemens et al. 2013; Patzke et al. 2018; Khan et al. 2023).

In plants such as sugar beet, orthologs of AtTST2 proteins are involved in sucrose storage in the vacuole (Jung et al. 2015). While cassava storage roots did not reach sugar beet levels of sucrose content, even the small storage roots analyzed in this study reached around 7.5% sucrose compared to their total DM, which was higher than any other tissue and implies an active concentration of the disaccharide. Appropriately, all cassava orthologs of *AtTST1/2* showed the same pattern as sucrose and hexose abundance with a strong increase at DB (Fig. 8). By contrast, homologs of the vacuolar sucrose exporter *AtSUC4* (Schneider et al. 2012) and other vacuolar exporters showed the opposite expression profile (Fig. 8). Direct measurements of sugars through NAF also suggest that high levels of sugars are accumulated in vacuoles and plastids. Sucrose and the monosaccharides glucose and fructose had the same relative distribution across the compartments and were most abundant in vacuoles, closely followed by plastids, with the cytosol having considerably lower relative sugar levels (Fig. 9). This change in expression of tonoplast transporters was also accompanied by pronounced increase of transcripts encoding for proteins and protein complexes involved in vacuole acidification (especially V-type ATPases; Gaxiola et al. (2007)), likely necessary for maintaining the high levels of secondary active transport across the tonoplast. The apparent increase in cellular- and subcellular osmotic potential during storage root bulking was also associated with an increase in expression of *PIPs* and *TIPs*, which facilitate water inflow into the symplasm and vacuoles, respectively.

Taken together, subcellular compartmentation appears to be an important aspect of root bulking that has multiple implications on the storage organ's physiology. For once, the proposed efflux of osmolytes from the symplasm in the storage root specifically would create a more favorable pressure gradient towards it, increasing its specific sink strength, hence allowing for more carbon to be allocated to the rapidly growing storage organ (Münch 1930). Furthermore, the large size of storage parenchyma cells compared to regular xylem parenchyma is probably facilitated through rapid vacuolar swelling comparable to how tracheary elements are formed (Kaiser and Scheuring 2020). Lastly, the accumulation of osmotic substances, followed by the inflow of water into storage root vacuoles might have evolved as a water storage function to endure the dry season. Regardless, competition between sugar compartmentalization and its utilization in starch biosynthesis might arise. Direct evidence for this was found recently in Nigerian field trials where a strong negative correlation between sucrose, hexoses, and most other measured metabolites with potential osmotic function and dry matter content—itself mainly being attributed to starch concentration—was observed (Gutschker et al. 2023; Lamm et al. 2023), indicating that this trait is not yet fixed in at least this particular cassava population, making it an interesting research and improvement target, even outside conventional breeding.

## Materials and methods

### Plant material

Cassava (*M. esculenta*) plants (genotype TME7) were planted in 14 l pots from 20 cm long stakes. Five plants were harvested at 5 timepoints: 25, 30, 38 (PB), 51 (EB), and 60 (DB) dap. Three PB, 5 EB, and 4 DB plants could be verified through microscopy to be in the desired developmental stage. The third to fifth source leaf without a middle vein, the wooden lower stem, the developing storage roots, as well as an assortment of side- and other fibrous roots were sampled. The lower stem was further split into bark and wood by peeling. Storage root samples were defined as a few centimeter-long pieces of the 2 to 4 thickest, nodal-derived roots, proximal to the stake. The samples were taken in the afternoon around 4 PM. For each of the samples, a small subsample was put into 30% ethanol (v/v) microscopy (toluidine blue and iodine staining). The developmental stage was determined based on the morphology of the thickest part of the furthest developed storage root.

### Paraffin embedding and histology

Specimen were fixed in 3.7% formaldehyde (v/v)/5% acetic acid (v/v) solution in 50% ethanol (v/v) overnight in a vacuum chamber, infiltrated in a Leica TP20: 1.5 h 70% ethanol (v/v), 2× 1.5 h 90% ethanol (v/v), 1 h 0.5% Eosin-Y (w/v) in 100% ethanol, 2× 1.5 h 100% ethanol, 2× 1.5 h 100% xylol, and 2× 24 h 100% paraffin (ParaplastTM Plus, 56 °C melting point) and embedded in a Leica EG1160. A Leica RM2265 was used for sectioning. Paraffin was removed using Roti-HistolTM for 20 min for 2 changes. The sections were then rehydrated in an ethanol series [100%, 100%, 90%, 80%, 70%, 50%, distilled water (v/v)] for 2 min each. Staining was performed using toluidine blue (0.1%, w/v) and iodine (5%, w/v).

### Metabolite measurements

Metabolite profiles, as well as sugar and starch measurements, were performed exactly as previously described (Rosado-Souza et al. 2019). For the sugar and starch extraction, the alternate method using perchloric acid was used.

### RNA sequencing and raw data processing

RNA was extracted using the Spectrum Plant Total RNA-Kit (Sigma-Aldrich). DB storage root samples were vortexed for 10 min rather than heated to 65 °C. RNA was sent to a service provider for paired-end mRNA sequencing (>20 million reads, PE150). The resulting FastQ files were quality-controlled using FastQC v0.11.9 (http://www.bioinformatics.babraham.ac.uk/projects/fastqc/) and MultiQC v1.13 (https://multiqc.info/). The reads were trimmed for adapter content and quality via bbduk v38.97 (http://sourceforge.net/projects/bbmap/). Bases under a quality of 30 were trimmed from both sides. Reads shorter than 35 base pairs or with an average quality below 30 after trimming were removed. The trimmed reads were mapped to the cassava genome v8.1. (including plastid and mitochondrial genome) with STAR v2.7.10a (Dobin et al. 2013). Mapped reads were counted using FeatureCounts v2.0.3 (Liao et al. 2014). Only uniquely mapped reads were counted. Multi-overlapping reads were not counted. The expression data for the storage roots was reproduced in an independent RNA-Seq experiment to ensure that these changes really reflect the root bulking process.

### Data analysis

Counts were normalized and log transformed (variance stabilization transformation, VST) using the R package DESeq2 v1.38.3 (Love et al. 2014). The DEG analysis against the developmental stage was performed for each tissue individually utilizing an LRT in DESeq2 using the intercept-only model as a comparison. Genes with FDR < 0.001 were accepted as DEGs. For the combined analysis of the 2 storage root experiments shown in Fig. 8, the VST values were batch-corrected using LIMMA v3.54.1 and the LRT executed with the experimental batch included as covariate. Downstream analyses were performed on the scaled and centered VST values. UMAP projection was performed in Python using the umap-learn module v0.5.3. DEGs were clustered through Louvain community detection with a resolution of 0.75 (Blondel et al. 2008) in python using the NetworkX module v3.0. Edges were drawn between genes based on a Pearson correlation coefficient above a permutation-based threshold (median 99.995% quantile of 1,000 replications). Genes < 50 edges were removed. For NSC data, generalized linear models were generated based on a Gamma distribution with a log link in base R. An ANOVA with type II sum of squares was executed using the car package. All *P*-values were adjusted for multiple testing using the FDR approach. Relative NAF results were tested using a linear model. Tukey's HSD was used as post-hoc test for significant results (FDR < 0.05). If not stated otherwise, plots were generated in R using the ggplot2 package and figures were prepared in Sketch for macOS.

### Functional annotation

Peptides from the cassava genome v8.1 and black cottonwood (*P. trichocarpa*) genome v4.1 were locally blasted (blast+ v2.13.0) against the thale cress (*A. thaliana*) proteome from the araport11 genome (e < 0.001). A list of aliases was downloaded from TAIR (arabidopsis.org, accessed on April 7 2021). Protein sequences were scanned against the Pfam database v35.0 using hmmer v3.3.2.

### Phylogenetic analysis

For each protein family, corresponding thale cress genes were selected via keyword search on arabidopsis.org. Suitable cassava and black cottonwood genes were selected based on BLASTP similarity to thale cress and the presence of a specified Pfam domain (Table 1). Full-length CDS were aligned using the auto option in MAFFT v7.511 (Katoh et al. 2002) and the alignments trimmed using TrimAl v1.4 (Capella-Gutiérrez et al. 2009). Genes with more than 50% gaps after trimming were removed and the alignment repeated using the l-ins-I algorithm. Maximum likelihood trees were generated using IQ-TREE v1.6.12 (Nguyen et al. 2014) with 1,000 ultra-fast bootstrap repetitions (Hoang et al. 2017) and Shimodaira–Hasegawa approximate-LRT (1,000 repetitions). The substitution model was selected using ModelFinder (Kalyaanamoorthy et al. 2017). Trees were drawn using the R packages ggtree. A full list of annotated genes can be found in Supplementary Data Set 3.

### Serial block face scanning electron microscopy

Leaves harvested from mature cassava were cut into 2 mm × 2 mm pieces and fixed in 4% glutaraldehyde, 2 mM CaCl2, 0.1 M cacodylate buffer, pH 6.8 at room temperature (RT) for 6 h. The samples then were fixed in a microwave oven (Biowave Pro, Pelco, Fresno CA, USA) at 350 W at 35 °C for 2 min. The leaves were washed 3 times 10 min with 0.1 M cacodylate buffer and then post-fixed overnight at 4 °C in 3% potassium ferricyanide, 0.3 M cacodylate, pH 6.8, 4 mM CaCl_2_, with an equal volume of 4% OsO_4_. The following day the samples were washed 3 times 10 min in ddH_2_O at RT, incubated for 40 min in 0.5% (w/v) thiocarbohydrazide, and washed again 3 times 10 min in ddH_2_O at RT. The samples were incubated in 2% OsO_4_ at RT and washed 3 times 10 min in ddH_2_O after 3 h. Samples were dehydrated in 10% increments of aceton from 0% to 100% and 3× 100% at RT 10 min each step. Following the dehydration, the samples were infiltrated in Hard Spurrs resin without DMAE as follows (1 part resin: 3 parts of acetone overnight, 1:2, 1:1, 2:1, 3:1, 100%, 2× 100% hard Spurrs with DMAE) and microwaved for 1 h at 100W at 40 °C max and cured for 48 h at 60 °C. The blocks were trimmed and mounted as described in Arcalís et al. (2020). Once the block was mounted on an SEM stub, sections were imaged on an Apreo Volumescope SEM (Thermo Fischer Scientific, USA). Image stack alignments, scaling, and adjustments were performed with Avizo software (Thermo Fisher Scientific) and the brightness/contrast was adjusted with ImageJ. Image processing, rendering, and animations were generated with Avizo software.

### Non-aqueous fractionation

A total of 8 to 10 mg of freeze-dried storage root material were used for NAF analyses based on a modified method of Fürtauer et al. (2016). A gradient of tetrachloroethylenes and n-heptanes with a total density of 1.35 to 1.60 (1.35; 1.40; 1.45; and 1.50 and 1.60) was mixed to suspend the freeze-dried samples. After sonication (1 h) in the lowest density gradient, samples were filtered and fractionated consecutively by centrifugation. Resulting supernatant was transferred to new tubes and used for the determination of marker enzyme activities and sugar levels measured. The resulting pellet was subsequently sonicated again in the gradient at the next higher density (20 min), filtered, and fractionated again via centrifugation. Determination of different activities of marker enzymes from vacuole, cytosol, and plastid was performed according to Fürtauer et al. (2016). To quantify the sugars from the different fractions, samples were first taken up in 300 *µ*l of H_2_O. For further analysis, 60 *µ*l of the sample was used and analyzed by NADP-coupled enzyme assay in 96-well plates via Tecan plate reader (Tecan Infinite 200, Tecan Group). The sugar levels and enzymatic activities are listed in Supplementary Data Set 7.

### Phloem exudates

The fifth leaf from the top was used for analysis. From such leaves, the fourth leaflet counted clockwise from the petiole was excised using a sharp razor blade. Leaflets were cut again while submerging the cutting site in 5 mM EDTA pH 8.0. Leaflets were shortly washed in 5 mM EDTA pH 8.0 to remove wounding sap. Exudates were then collected, as described in Pommerrenig et al. (2007). Exudates were collected for 10 h in 500 *µ*l of 5 mM EDTA pH 8.0 solution. FWs of leaflets were determined after exudation and used for the calculation of obtained sugar amounts. Sugars in the exudates were determined using ion chromatography, as described below.

### Determination of soluble sugars from phloem exudates

A 20 *µ*l of the phloem exudate were directly used for sugar determination. Glucose, fructose, sucrose, and raffinose were measured with ion chromatography, as described by Ho et al. (2020). Briefly, a 761 Compact IC system (Metrohm, Herisau, Switzerland) was used. A Metrosep Carb2-250/4.0 column was used in combination with a Metrosep Carb2 Guard/4.0 guard column (both Metrohm, Herisau, Switzerland) followed by amperometric quantification. The mobile phase consisted of NaOH (100 mM) and sodium acetate (10 mM). Peak analysis was done using Metrodata IC Net 2.3 SR5 software by Metrohm.

### Accession numbers

All accession numbers analyzed in this study can be found in Supplementary Data Set 3.

## Supplementary Material

kiae298_Supplementary_Data

## Data Availability

All used commands and scripts are available on github (https://github.com/Division-of-Biochemistry-Publications/CASS). Raw sequencing reads have been deposited to NCBI's Sequence Read Archive under BioProject ID PRJNA1045511, available at https://www.ncbi.nlm.nih.gov/bioproject/PRJNA1045511.

## Supplementary data

The following materials are available in the online version of this article.

**Supplementary Figure S1.** Lugol staining of stem and storage root tissues.

**Supplementary Figure S2.** Sucrose concentration in cassava source leaves.

**Supplementary Figure S3.** Sugars in cassava phloem exudates.

**Supplementary Data Set 1.** Non-structural carbon measurements for the different tissues and timepoints.

**Supplementary Data Set 2.** Clustering results of DEGs for each tissue.

**Supplementary Data Set 3.** Phylogenetically annotated *M. esculenta* genes of this study.

**Supplementary Data Set 4.** Results of phloem exudate measurements.

**Supplementary Data Set 5.** Results of leaf sugar measurements based on fresh weight.

**Supplementary Data Set 6.** Student's *t*-test for each metabolite and tissue between EB and DB for all tissues.

**Supplementary Data Set 7.** Raw data of the NAF measurements.

**Supplementary File 1.** Treefile containing the phylogenetic tree of *A. thaliana*, *M. esculenta*, and *P. trichocarpa AATP* genes.

**Supplementary File 2.** Treefile containing the phylogenetic tree of *A. thaliana*, *M. esculenta*, and *P. trichocarpa Acid INV* genes.

**Supplementary File 3.** Treefile containing the phylogenetic tree of *A. thaliana*, *M. esculenta*, and *P. trichocarpa A/N INV*genes.

**Supplementary File 4.** Treefile containing the phylogenetic tree of *A. thaliana*, *M. esculenta*, and *P. trichocarpa AQP* genes.

**Supplementary File 5.** Treefile containing the phylogenetic tree of *A. thaliana*, *M. esculenta*, and *P. trichocarpa MST* genes.

**Supplementary File 6.** Treefile containing the phylogenetic tree of *A. thaliana*, *M. esculenta*, and *P. trichocarpa PPASE* genes.

**Supplementary File 7.** Treefile containing the phylogenetic tree of *A. thaliana*, *M. esculenta*, and *P. trichocarpa SUS/SPS* genes.

**Supplementary File 8.** Treefile containing the phylogenetic tree of *A. thaliana*, *M. esculenta*, and *P. trichocarpa SUT* genes.

**Supplementary File 9.** Treefile containing the phylogenetic tree of *A. thaliana*, *M. esculenta*, and *P. trichocarpa SWEET* genes.

**Supplementary File 10.** Treefile containing the phylogenetic tree of *A. thaliana*, *M. esculenta*, and *P. trichocarpa TPT* genes.

**Supplementary File 11.** Treefile containing the phylogenetic tree of *A. thaliana*, *M. esculenta*, and *P. trichocarpa V-ATPase* genes.

## Dive Curated Terms

The following phenotypic, genotypic, and functional terms are of significance to the work described in this paper:

SUS1 Gramene: AT5G20830
SUS1 Araport: AT5G20830

INT1 Gramene: AT2G43330
INT1 Araport: AT2G43330

phloem AmiGo: PO:0005417

myo-inositol CHEBI: CHEBI:17268

SUS3 Gramene: AT4G02280
SUS3 Araport: AT4G02280

EDTA Gramene: Ethylenediamine tetraacetic acid
EDTA Araport: Ethylenediamine tetraacetic acid

metabolite CHEBI: CHEBI:25212

TPT Gramene: At5g46110
TPT Araport: At5g46110

transfer cell AmiGo: PO:0000078
