# Supplementary material for: Symplasmic phloem loading and subcellular transport in storage roots are key factors for carbon allocation in cassava
Source: Plant Physiol. 2024 May 22;196(2):1322–39. doi: 10.1093/plphys/kiae298 (PMC11483629; doi:10.1093/plphys/kiae298)
Supplement: kiae298_Supplementary_Data [file kiae298_Supplementary_Data.zip › PP2024RA00276R1Supplementary Figures.pdf]

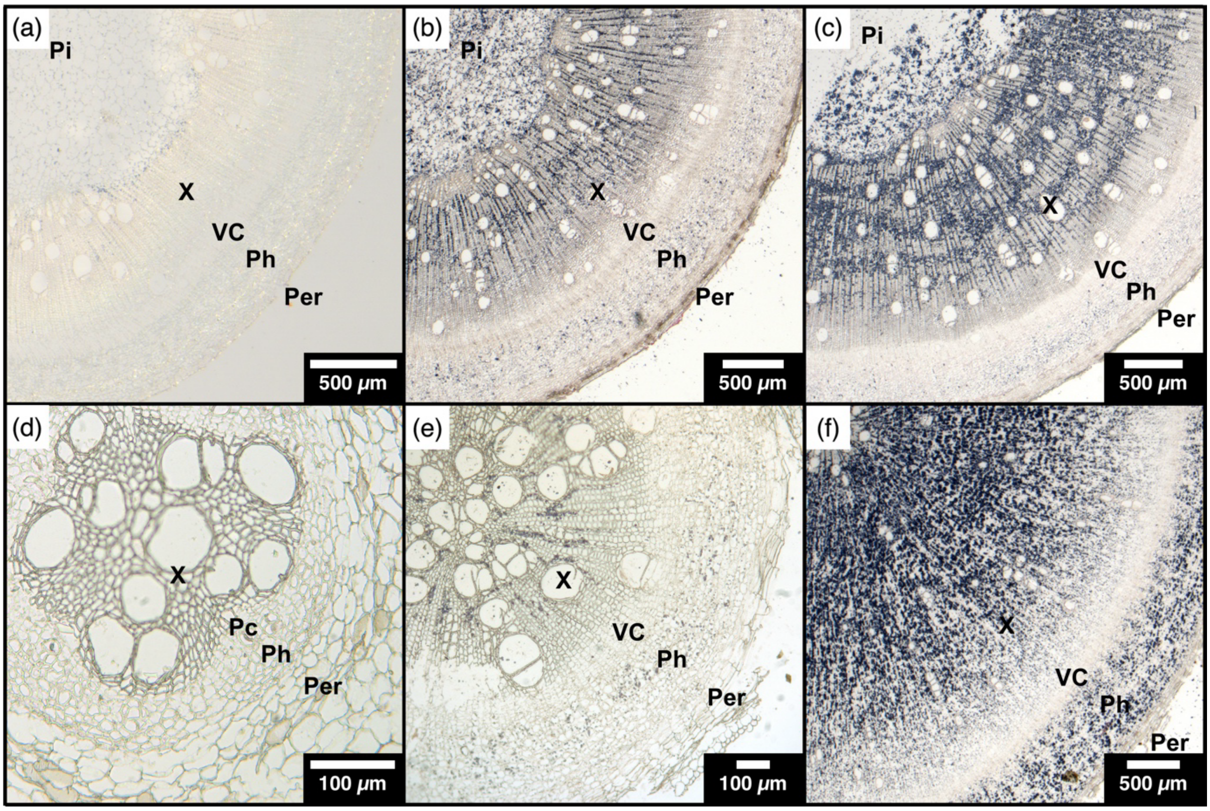

Supplementary Figure S1. Lugol staining of stem and storage root tissues.
(a-c) Micrographs of paraffin-embedded stem cross-sections of the pre-bulking stage (a), early-bulking (b), and during bulking stage (c), which were each stained with Lugol solution to visualize starch granules. (c), (f): Identical images were also used in Figure 2e-f. Abbreviations: Per: periderm, Ph: phloem, Pi: pith, VC: vascular cambium, X: xylem

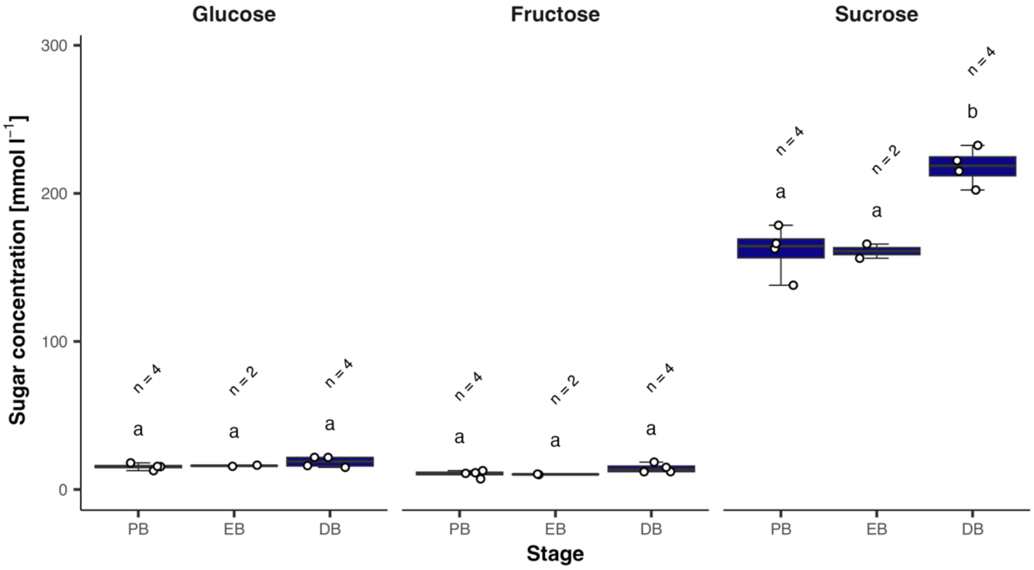

Supplementary Figure S2. Sucrose concentration in cassava source leaves. Columns from left to right show glucose, fructose, and sucrose of source leaves in millimole per liter of as scatter plots (white dots) and standard boxplots (line: median, box: Quartile 1 to 3, whiskers: minimum and maximum value excluding outliers). Sugars were measurement in fresh samples. Water content was determined in a separate aliquot of the same samples. A generalized linear model was fitted on the raw values versus the stage for each tissue. Letters show groups of significance within each sugar (Tukey HSD $p \leq 0.05$) of models with an FDR adjusted p value < 0.05 in an ANOVA. Abbreviations: PB: Pre-bulking, EB: Early-bulking, DB: During-bulking.

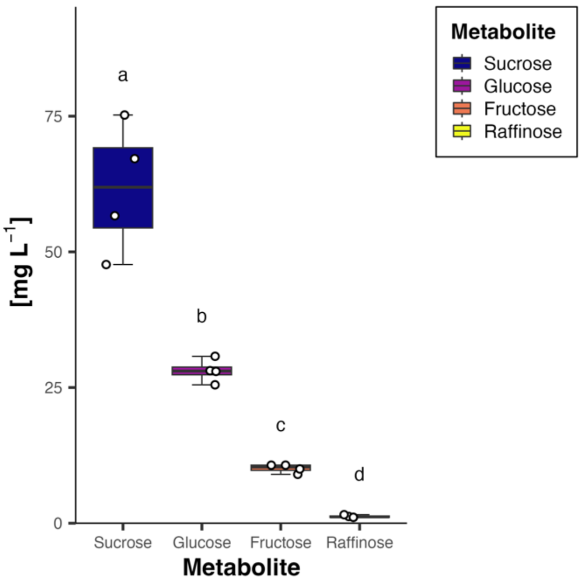

Supplementary Figure S3. Sugars in cassava phloem exudates.

Sucrose, glucose, fructose, and fructose content of leaf phloem exudates are shown in milligram per gram of dry weight as scatter plots (white dots) and standard boxplots (line: median, box: Quartile 1 to 3, whiskers: minimum and maximum value excluding outliers). A generalized linear model was fitted on the raw values versus the different metabolites. Letters show groups of significance (Tukey HSD $p \leq 0.05$) of models with an FDR adjusted p value < 0.05 in an ANOVA.
