# Supplementary material for: Symplasmic phloem loading and subcellular transport in storage roots are key factors for carbon allocation in cassava
Source: Plant Physiol. 2024 May 22;196(2):1322–39. doi: 10.1093/plphys/kiae298 (PMC11483629; doi:10.1093/plphys/kiae298)
Supplement: kiae298_Supplementary_Data [file pp.00276.2024-s22.pdf › pp.00276.2024-s22.pdf]

Plant Physiology®

Symplasmic phloem loading and sub-cellular transport in storage roots are key factors for carbon allocation in cassava

David Rüscher, Viktoriya Vasina, Jan Knoblauch, Leo Bellin, Benjamin Pommerrenig, Saleh Alseekh, Alisdair Fernie, H. Neuhaus, Michael Knoblauch, Uwe Sonnewald, and Wolfgang Zierer

Review Timeline:

Submission Date:	21-Feb-2024
Editorial Decision:	05-Apr-2024
Revision Received:	30-Apr-2024
Accepted:	05-May-2024

05-Apr-2024

Dr. Wolfgang Zierer
Friedrich-Alexander University Erlangen-Nuremberg
Erlangen, Bavaria
Germany

RE: Symplasmic phloem loading and sub-cellular transport in storage roots are key factors for carbon allocation in cassava

Dear Dr. Wolfgang Zierer:

We are pleased to accept your manuscript for publication in the Plant Physiology. This acceptance is contingent on revision based on the comments of our reviewers. In particular, please consider the following:

Please address all of the issues and suggestions raised by the two reviewers. Attention to these concerns will, in my opinion, result in a stronger paper!

Please highlight all changes and include a detailed annotation to changes to the text, with line numbers, and noting your responses to the comments.

To submit your revised manuscript, click:

Link Not Available

If you cannot return the revised manuscript within 8 weeks of receipt, please let us know. Otherwise, we will assume that you have elected not to revise the manuscript and are withdrawing it.

Thank you for allowing us to review your work. We look forward to hearing from you soon.

Sincerely,

Editor
Plant Physiology

-- Reviewer comments:
Reviewer #1 (Comments for the Author):

The MS extends the work of the authors on the pathway of assimilate transport into the storage tap root of cassava. The data showing are a comprehensive analysis of expression levels in source leaves, stem and storage root at three different developmental stages. By combining these data, the MS casts light on the orchestration of photosynthetic carbohydrate production, transport and storage. The serial block face 3D electron microscopy and the supplementary video are excellent to visualize the different cell bridges in minor veins of cassava source leaves.
The MS is generally well written, though the result part is not concise and would benefit from shortening and moving references to Introduction or Discussion (e.g., lines 115-202; lines 276-282). Fig. 7 is a combination of showing the different expression profiles of the studied genes with heat maps of the expression levels, and at the same time suggesting a model.
I am aware of the fact that comprehensive data on transcriptomics are challenging to communicate using Figures but in my opinion, the model would fit much better in the discussion giving the authors' interpretation of the acquired data and the conclusion the reader can take home from the MS.
The authors conclude that cassava loads and unloads assimilates symplasmically. This conclusion is derived from the seeming lack of SWEET facilitators and SUT1/SUC2 sucrose-proton co-transporters in leaves, and from the existence and abundance of plasmodesma connections between bundle sheath and/or parenchyma cells and companion cell of minor veins. Existence of plasmodesmatal structures cannot be taken as evidence for their functionality. Stronger evidence for the claim made in the discussion and emphasized as subheading 'Parenchymatic cells of source and sink tissues are symplasmically connected (line 358) would be provided exploiting tracer experiments.This was done before only for the unloading pathway from the phloem to the rays (Mehdi et al. 2019).
Minor points:
line 39: 'high sucrose concentrations in leaves' Fig. 3 shows sucrose per DW for entire leaves - how does this relate to leaf concentrations and to the list, Rennie and Turgeon have provided as typical for symplasmic loaders?
line 120-121: 'The storage root did not produce many tracheary elements and almost no fibers (Fig. 2c), while the lower stem

displayed the opposite phenotype' _phenotype_- is normally related to genetics, here 'morphology' would be more appropriate.
line 139 'rapidly accumulated' Since the two stages are defined with a a 9-day gap (see lines 103-104), the strong accumulation observed in the DB stage cannot be considered as happening _rapidly_!
line 142. 'subsequently dropped' no statistically significant reduction!
line 203: 'Expression of both MeSUT1 orthologs decreased over time in the storage root and bark but increased in the wood (Fig. 5d)' not visible in the Fig. because of overlapping symbols.
line 211: 'Several _symmetrically_ branched plasmodesmata were present at the CC/BS interface (Fig. 6a, c)' As far as I could check this in the video, they are branched on either cell wall side. But the branching is not symmetrical, since the branching point seems to be much closer to the bundle sheath than to the CC in all cases.
Line 495 and 498: 'symplast' The noun matching the adjective symplasmic is symplasm, not symplast (see cytoplasm)

Fig. 2 caption: 'Stereo micrographs' rather 'Image pairs'; a stereo micrograph would show a 3D view of a tissue by separating two view angles through red/green or polar filters.
Fig. 3 caption: ' Letters show groups of significance within each _facet_' rather 'panel'
Fig. 4 caption: do not use abbreviations, explain UMAP, SESeq2, DEGs, and FDR for non-bioinformatic readers!
Fig. 5d. add 'a' and 'b' to the upper and lower SUT1 heading, respectively.
Fig. 6. Excellent micrographs, but please add a one-plane cross section for each, d, e, and f. and below that each of the present panels. As it is now, the minor vein architecture cannot really be appreciated, since it is covered by the animated 3D projection.
Fig. 7: See my comment on data/ model above. Moreover, I'd suggest in addition to use straight lines only for plasma membranes of all cells, and place the ellipses centrally on the respective plasma membrane. At the moment they are placed on the middle lamella e.g. between ray and tracheary element in stem and SR. Use white lettering on the dark violet and red ellipses; cannot be read in the present version. It is unclear to which enzymes/transporters the heat maps refer to, e.g., only the result text tells me that SUS11 and SUS1b is the upper one of the heat map box in SR. Which three SUS5s are meant in the heat map box of the phloem with totally different expression pattern in stem and SR? Which four vacuolar SWEETs are meant inf the xylem parenchyma vacuoles?
Fig. 8: Same here: please provide labels for the heat map boxes of all different members s of a protein family,
Material and methods
Line 538-540 For the carbohydrate analysis (Fig. 3) you refer to the method article Rosado-Souza et al. 2019. Please give anyway a short description what you did in the present article! The cited paper provides two protocols Basic protocol 3 and alternate protocol with perchloric-acid extraction. I guess that you used the alternate protocol - but this should be spelled out.

Reviewer #2 (Comments for the Author):

This is a detailed study of phloem loading, unloading and sink development in Cassava that provides important insights into carbon allocation in these valuable plants. By combining high quality histological work with transcriptomics and work on sugar compartmentalization, the study presents an impressive and thorough analysis of phloem physiology in this species. I think the study would be of high interest not only to researchers interested in Cassava but also the broader physiological community because it provides detailed information on phloem physiology, phloem loading and sink development in non-model plant. The paper is clearly written with well-designed figures. I only have a handful of small comments below.

- This paper focuses heavily on Cassava and might benefit from placing some of the research in a broader context. Based on my understanding, there are not many species that we have this type of detailed phloem/metabolite/transcriptome data on. If there are novel aspects to this work when framed in the context of broader questions in plant physiology, it might be nice to highlight them in the abstract/intro/discussion. It might help the paper attract a broader audience.

- Data from phloem exudates are presented in Fig. S2 but I did not see any description of how and where exudates were collected in the methods.

- In Fig. 7 what is PS?

Dear Reviewers,

Thank you very much for investing time and effort into the improvement of our manuscript and for providing largely positive and constructive feedback! Please find our responses in black writing below.

On behalf of the authors,
Wolfgang Zierer

Reviewer 1

General comments

The MS extends the work of the authors on the pathway of assimilate transport into the storage tap root of cassava. The data showing are a comprehensive analysis of expression levels in source leaves, stem and storage root at three different developmental stages. By combining these data, the MS casts light on the orchestration of photosynthetic carbohydrate production, transport and storage. The serial block face 3D electron microscopy and the supplementary video are excellent to visualize the different cell bridges in minor veins of cassava source leaves. The MS is generally well written, though the result part is not concise and would benefit from shortening and moving references to Introduction or Discussion (e.g., lines 115-202; lines 276-282). Fig. 7 is a combination of showing the different expression profiles of the studied genes with heat maps of the expression levels, and at the same time suggesting a model. I am aware of the fact that comprehensive data on transcriptomics are challenging to communicate using Figures but, in my opinion, the model would fit much better in the discussion giving the authors' interpretation of the acquired data and the conclusion the reader can take home from the MS. The authors conclude that cassava loads and unloads assimilates symplasmically. This conclusion is derived from the seeming lack of SWEET facilitators and SUT1/SUC2 sucrose-proton co-transporters in leaves, and from the existence and abundance of plasmodesma connections between bundle sheath and/or parenchyma cells and companion cell of minor veins. Existence of plasmodesmatal structures cannot be taken as evidence for their functionality. Stronger evidence for the claim made in the discussion and emphasized as subheading 'Parenchymatic cells of source and sink

tissues are symplasmically connected (line 358) would be provided exploiting tracer experiments. This was done before only for the unloading pathway from the phloem to the rays (Mehdi et al. 2019).

We thank the reviewer for these thoughtful comments. We repeatedly discussed the structure of the manuscript and agreed that some shorting can still be done. Consequently, we did shorten the morphological descriptions and moved the statement on root development to the introduction section. Also, we removed a small section that was redundant with the discussion from the results section. However, we would like to keep an overall thorough description of the materials used in this study, as we feel it is important for the context of the study. In addition, there are not many transcriptome datasets for different cassava tissues published, making these data and associated metadata a potentially valuable resource for other cassava researchers. While we like the idea of a discussion figure, we feel that without Fig. 7 the results on tissue-specific enzymes and subcellular transporters in leaves, stems and storage roots are much harder to communicate. For us the main function of Fig. 7 is less of a model, but more to illustrate the significant changes in tissue-specific gene expression across the three developmental stages. Therefore, we would like to keep the current order of figures, but renamed Fig. 7 to "Changes in tissue-specific gene expression for annotated genes in leaf, stem, and storage root" and remove the reference to a model in the text.

We fully agree that we cannot unambiguously proof that cassava is a passive symplasmic phloem loader and that the mere presence of plasmodesmata is no definitive proof for their functionality. Unfortunately, tracer experiments for phloem loading are technically very challenging and also somewhat prone to artefacts. Ideally, we would also need a *mesuc2* knockout plant, which would require years of work.

We think that we were reasonably careful in the manuscript, also stating that there is never a black and white scenario for phloem transport and that there is likely also at least some active transport. However, the sum of indications collected in this study (the incredibly high sucrose levels in source leaves, the presence of PDs in the CC-BS interface, the lack of *SWEET* expression specifically in leaves, the general expression pattern of *SUC2* being similar to tree species) makes it very likely, in our opinion, that cassava employs a largely passive symplasmic phloem loading.

Minor points

line 39: 'high sucrose concentrations in leaves' Fig. 3 shows sucrose per DW for entire leaves - how does this relate to leaf concentrations and to the list, Rennie and Turgeon have provided as typical for symplasmic loaders?

We wanted to illustrate the tissue-specific changes of NSCs in the different tissues and timepoints in Fig. 3 (and therefore used tissue dry weight as basis for calculations). The high sucrose concentration in source leaves is shown in the Supplementary Material Fig. S3 on the basis of fresh material as mentioned in the results section ("In addition, the sucrose concentration in cassava source leaves is very high with more than 200 mmol l^{-1} at the DB stage (Fig. S3; Supplementary file 16), which is another characteristic of passive symplasmic loaders (Rennie and Turgeon, 2009)"). Rennie and Turgeon report the same units and their highest passive symplasmic loader has 150 mmol l^{-1}. We added the sentence "The highest sucrose concentration reported there was 150 mmol l^{-1} for a passive symplasmic loader." to make this point clearer.

- line 120-121: 'The storage root did not produce many tracheary elements and almost no fibers (Fig. 2c), while the lower stem displayed the opposite phenotype' _phenotype_- is normally related to genetics, here 'morphology' would be more appropriate.

We have changed this to "morphology".

- line 139 'rapidly accumulated' Since the two stages are defined with a a 9-day gap (see lines 103-104), the strong accumulation observed in the DB stage cannot be considered as happening _rapidly_!

We deleted the word "rapidly".

- line 142. 'subsequently dropped' no statistically significant reduction!

We deleted the statement.

- line 203: 'Expression of both *MeSUT1* orthologs decreased over time in the storage root and bark but increased in the wood (Fig. 5d)' not visible in the Fig. because of overlapping symbols.

We agree that the visibility is not 100% ideal in this figure. However, changing the overall layout of the graphs would require much more space. To make it more readable we increased the Y-dimension of the Fig. 5d.

- line 211: 'Several _symmetrically_ branched plasmodesmata were present at the CC/BS interface (Fig. 6a, c)' As far as I could check this in the video, they are branched on either cell wall side. But the branching is not symmetrical, since the branching point seems to be much closer to the bundle sheath than to the CC in all cases.

We removed the characterization of the plasmodesmata branching.

- line 495 and 498: 'symplast' The noun matching the adjective symplasmic is symplasm, not symplast (see cytoplasm)

We changed this accordingly.

- Fig. 2 caption: 'Stereo micrographs' rather 'Image pairs'; a stereo micrograph would show a 3D view of a tissue by separating two view angles through red/green or polar filters.

We changed this to “Image pairs”.

- Fig. 3 caption: ' Letters show groups of significance within each _facet_' rather 'panel'

We changed this to “panel”.

- Fig. 4 caption: do not use abbreviations, explain UMAP, SESeq2, DEGs, and FDR for non-bioinformatic readers!

We simplified the caption to make it more understandable. However, a more detailed explanation of these bioinformatic methods/algorithms is beyond the scope of the manuscript.

- Fig. 5d. add 'a' and 'b' to the upper and lower SUT1 heading, respectively.

We added “a” and “b” to the upper and lower SUT1 heading.

- Fig. 6. Excellent micrographs, but please add a one-plane cross section for each, d, e, and f. and below that each of the present panels. As it is now, the minor vein architecture cannot really be appreciated, since it is covered by the animated 3D projection.

We are not sure we fully understand this comment. Fig. 6a is already a one-plane cross-section of the minor vein, with zoom-ins for the SE-CC (Fig. 6b) and CC-BS (Fig. 6c) interface. We separated the individual pictures by boxes and distances to differentiate them more clearly.

- Fig. 7: See my comment on data/ model above. Moreover, I'd suggest in addition to use straight lines only for plasma membranes of all cells, and place the ellipses centrally on the respective plasma membrane. At the moment they are placed on the middle lamella e.g. between ray and tracheary element in stem and SR. Use white lettering on the dark violet and red ellipses; cannot be read in the present version. It is unclear to which enzymes/transporters the heat maps refer to, e.g., only the result text tells me that SUS11 and SUS1b is the upper one of the heat map box in SR. Which three SUS5s are meant in the heat map box of the phloem with totally different expression pattern in stem and SR? Which four vacuolar SWEETs are meant inf the xylem parenchyma vacuoles?

We have improved the layout of Fig. 7 according to the reviewer´s suggestions and added the respective gene information.

- Fig. 8: Same here: please provide labels for the heat map boxes of all different members s of a protein family,

We have improved the layout of Fig. 8 according to the reviewer´s suggestions and added the respective gene information.

Material and methods

- Line 538-540 For the carbohydrate analysis (Fig. 3) you refer to the method article Rosado-Souza et al. 2019. Please give anyway a short description what you did in the present article! The cited paper provides two protocols Basic protocol 3 and alternate protocol with perchloric-acid extraction. I guess that you used the alternate protocol - but this should be spelled out.

We indicated the use of the alternate protocol.

Reviewer 2

Minor points

This is a detailed study of phloem loading, unloading and sink development in Cassava that provides important insights into carbon allocation in these valuable plants. By combining high quality histological work with transcriptomics and work on sugar compartmentalization, the study presents an impressive and thorough analysis of phloem physiology in this species. I think the study would be of high interest not only to researchers interested in Cassava but also the broader physiological community because it provides detailed information on phloem physiology, phloem loading and sink development in non-model plant. The paper is clearly written with well-designed figures. I only have a handful of small comments below.

- This paper focuses heavily on Cassava and might benefit from placing some of the research in a broader context. Based on my understanding, there are not many species that we have this type of detailed phloem/metabolite/transcriptome data on. If there are novel aspects to this work when framed in the context of broader questions in plant physiology, it might be nice to highlight them in the abstract/intro/discussion. It might help the paper attract a broader audience.

We thank the reviewer for this interesting suggestion. We think that the phloem results for the shrub cassava are similar to the results obtained from tree species, but quite different to the results from other model plants or crop plants, often using a more apoplasmic mode of transport. We did mention this aspect in the manuscript. Storage roots and storage organs (beyond maybe potato) are unfortunately still quite under researched. We absolutely agree that more detailed studies on other crop plants would be very interesting, but would like to refrain from speculating about other plants where we have not data on.

- Data from phloem exudates are presented in Fig. S2 but I did not see any description of how and where exudates were collected in the methods.

We thank the reviewer for pointing this out. We did indeed overlook this method and now added it to the Material and Methods section.

- In Fig. 7 what is PS?

PS was used to indicated Photosynthesis.

05-May-2024
Dr. Wolfgang Zierer
Friedrich-Alexander University Erlangen-Nuremberg
Erlangen, Bavaria
Germany

MSID: PP2024-RA-00276R1
MS TITLE: Symplasmic phloem loading and sub-cellular transport in storage roots are key factors for carbon allocation in cassava

Dear Dr. Wolfgang Zierer:

I am pleased to inform you that your revised manuscript "Symplasmic phloem loading and sub-cellular transport in storage roots are key factors for carbon allocation in cassava" has been accepted for publication in Plant Physiology. Your manuscript will be checked for consistency with journal formatting and image standards by one of our Science Editors, who will contact you shortly. They may have a number of queries and suggestions for you. Please keep in mind that they work to increase the online visibility of your article and its attraction for readers from the widest of backgrounds. You will have the opportunity to review any revisions, and I hope you will work constructively with them to get the most out of your research. You will also have the opportunity to review all changes when you receive the author proofs for your article.

Once validated for publication, your article will be sent to a Science Editor for a final review of your manuscript and source files. After your final files are uploaded, your article will appear in the next available issue and the online Preview version of your article will be posted after your signed Copyright and Checklist forms and final source files are received.

Please be sure to include the following in your manuscript file:
Harvard style citations
List of all author contributions and funding information at the beginning of manuscript after the titles and author list.
List all supplemental materials associated with the article, with titles, at the end of the ms, before the references and acknowledgments section.
Complete Figure Legends for all figures appearing in the article listed before the Literature Cited.

Thank you and congratulations on your Plant Physiology paper!

Sincerely,
Editor
Plant Physiology

=====
FIRST AUTHOR PROFILES
=====

We recognize the hard work that goes into being First Author of a Plant Physiology paper by publishing a "First Author Feature" for each First Author alongside your paper.

If the first author(s) would like to submit information for a feature on Facebook and Twitter, fill out the form by clicking on the link below:

Plant Physiology First Author Feature Form: https://forms.office.com/r/ZBCGZfhm8F
If you cannot access the form through the link above, please contact Rachel at rbelsky@aspb.org.

To upload an image of yourself to accompany your profile, please put your photo in the correct journal folder at the link below. You must label your image: Author First Name Last Name Article Title.

First Author Profile Image Dropbox: https://www.dropbox.com/scl/fo/xz029p7qjr2jml2rp2kmq/h?dl=0&rlkey=6fkxcaqmxx35ap152b2osy1fz

=====
IMPORTANT REMINDER: PEER REVIEW REPORTS
=====

If you opted to publish a peer review report along with your article during the original submission process, it will be prepared by the editorial staff and publicly posted with your manuscript, inside the zip file that contains any other supplemental material. As a reminder, the peer review report is a public record of all comments from editors and reviewers, as well as your prior responses, as you received them in the decision letters for each draft of your manuscript. If you agreed to publish this report and have changed your mind, or are not sure if you selected this option, please contact the editorial office as soon as possible before signing the license agreement from our publisher.

-- Reviewer comments:
